# A vaccine-based nanosystem for initiating innate immunity and improving tumor immunotherapy

**DOI:** 10.1038/s41467-020-15927-0

**Published:** 2020-04-24

**Authors:** Di-Wei Zheng, Fan Gao, Qian Cheng, Peng Bao, Xue Dong, Jin-Xuan Fan, Wen Song, Xuan Zeng, Si-Xue Cheng, Xian-Zheng Zhang

**Affiliations:** Key Laboratory of Biomedical Polymers of Ministry of Education & Department of Chemistry, Wuhan University, Wuhan, 430072 PR China

**Keywords:** Breast cancer, Cancer therapy

## Abstract

The unsatisfactory response rate of immune checkpoint blockade (ICB) immunotherapy severely limits its clinical application as a tumor therapy. Here, we generate a vaccine-based nanosystem by integrating siRNA for *Cd274* into the commercial human papillomavirus (HPV) L1 (HPV16 L1) protein. This nanosystem has good biosafety and enhances the therapeutic response rate of anti-tumor immunotherapy. The HPV16 L1 protein activates innate immunity through the type I interferon pathway and exhibits an efficient anti-cancer effect when cooperating with ICB therapy. For both resectable and unresectable breast tumors, the nanosystem decreases 71% tumor recurrence and extends progression-free survival by 67%. Most importantly, the nanosystem successfully induces high response rates in various genetically modified breast cancer models with different antigen loads. The strong immune stimulation elicited by this vaccine-based nanosystem might constitute an approach to significantly improve current ICB immunotherapy.

## Abstract (web summary)

Immune checkpoint blockade (ICB) immunotherapy efficacy has still limitations. Here, the authors generate a vaccine that integrates CD274 siRNA into the L1 protein of human papillomavirus, which cooperates with ICB by activating innate immunity in breast cancer models.

## Introduction

Immunotherapy for unleashing patients’ autologous immune system has achieved clinical benefits in various incurable tumors^1–6^. Clinically, immune checkpoint blockade (ICB), especially the pharmaceutical targeting of programmed cell death protein 1 or programmed cell death protein 1(PD1/PDL1) has displayed satisfactory effects in treating patients with melanoma, non-small cell lung cancer and triple-negative breast cancer (TNBC)^7^. Despite these achievements, a primary problem facing ICB therapy in clinical trials is the extremely low response rate. Even in melanoma, one of the most immunogenic types of cancer, only 20–50% of patients benefit from ICB treatment^8^.

With the ability to elicit robust and durable immune responses, microorganisms have been used to boost the therapeutic response rate for cancer therapies^9–11^. Very recently, the intravenous (*i.v.*) injection of material-assisted or genetically modified *Escherichia coli* was demonstrated to activate innate immunity and suppress the tumor growth^12,13^. Importantly, the intratumoral injection of oncolytic virus, T-Vec, was found to increase the response rate of ipilimumab treatment in advanced melanoma patients for 2.9-fold^14^. However, the safety concern is the biggest problem for current microorganism-based therapy. For example, the adverse effects resulting from bacteria (e.g., cytokine release syndrome and infection) or viruses (mainly fatigue, fevers, and chills), much restrict their clinical translation.

Vaccines are biologically prepared infectious agents with the microorganism-like structure, and they are highly safe^15^. Evidence has proved that the current-used nonliving vaccines lead to robust immune responses. In a phase 2b–3 study, 9-valent human papillomavirus (HPV) vaccine reached a high protection rate of 96.7% in 14,215 women, and only 2% of subjects displayed mild systemic side effects (e.g., pain, swelling, erythema, and pruritus)^16^. Having been widely vaccinated in population and stood the test of time, vaccines share the advantages with guaranteed safety for full translational potential.

Encouraged by the microorganisms-like property and proper biosafety, we modifies a vaccine to improve the response rate of current ICB therapy. Considering the capacity of microorganisms in inducing immune responses, we analyzes the correlation between the expression of innate immunity-associated genes and clinical prognosis in various cancer types. From in silico analysis, breast cancer patients with innate immune activation are found to have a better prognosis. Based on this fact, TNBC, the most aggressive form of breast cancer, is selected for testing our therapeutic strategy. As one of the most widely used vaccines in the clinic, the HPV vaccine (HPV16 L1 protein assembly) is chosen and used for modification. Previously, immunotherapy targeting PDL1 has achieved excellent results in clinical trials of breast cancer patients. Utilizing the specific binding between HPV capsid and α_6_ integrin over-expressed in TNBC^17^, PEGylated HPV16 L1 protein is assembled with siRNA oligonucleotides (for *Cd274* knockdown) for inhibiting the expression of tumor-specific PDL1^18,19^. Benefiting from the advantages of the HPV vaccine, this siRNA loaded pseudovirus nanoparticles (siRNA@HPVP) induces effective immunotherapy effects with a high response rate and superior biosafety. The design of siRNA@HPVP establishes a convenient method for increasing the response rate of ICB therapy through the combination of the microbial-based vaccines in use.

## Results

### Construction of siRNA@HPVP

**Fig. 1:**
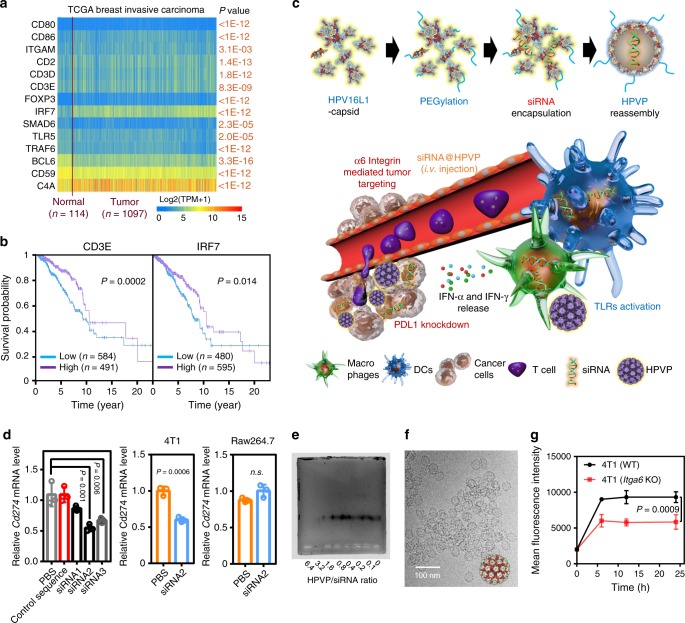
Schematic and characterization of siRNA@HPVP. **a** Expressions of tumor immune regulation-associated genes in 1097 human breast tumors and 114 para-carcinoma tissues from UALCAN (http://ualcan.path.uab.edu/index.html). Statistical significance was calculated with two-tailed Student’s *t* test. **b** The correlation between the *CD3E*/*IRF7* expressions and the survival of breast tumor patients from TCGA. Statistical significance was calculated with two-tailed Student’s *t* test. **c** Schematic diagram for the design and function process of the siRNA@HPVP system. Before the encapsulation of siRNA, the HPV16 L1 capsid was disassembled by DTT. Disassembled HPV16 L1 protein mixed with siRNA and PEG was reassembled into nanoparticles through the removal of DTT via dialysis. The HPVP was expected to initiate an immune response in an *Irf7*-dependent manner (mainly in APCs), while the knockdown of the *Cd274* gene (mainly in cancer cells) promoted the lymphocyte infiltration. **d** PCR analysis of *Cd274* transcription in 4T1 cells after silenced by three siRNA sequences (100  nmol mL^−1^). The gene silencing efficiency of siRNA2 on 4T1 cells and RAW 267.4 cells. Three biological replicates are shown. Statistical significance was calculated with one-way ANOVA with Tukey post-hoc (left) and two-tailed Student’s *t* test (middle and right). **e** Gel electrophoresis of siRNA@HPVP at different mass ratios between HPVP and siRNA, naked siRNA was set as control. **f** Cryo-TEM images of siRNA@HPVP. A representative image of two biological replicates is shown. **g** Flow cytometry data of the uptake of siRNA@HPVP in 4T1 cells or 4T1^*Itga6*-^ cells. The intracellular fluorescence intensity was measured at 6 h, 12 h, and 24 h of the co-incubation. Three biological replicates are shown. Statistical significance was calculated with two-tailed Student’s *t* test. Data are presented as mean values ± SD.

First, transcriptome and survival data were obtained from The Cancer Genome Atlas (TCGA) in silico analysis. The expression of a list of genes that associated with immune regulation (*CD80*, *CD86*, *ITGAM*, *CD2*, *CD3D*, *CD3E*, *FOXP3*, *IRF7*, *SMAD6*, *TLR5*, *TRAF6*, *BCL6*, *CD59*, and *C4A*) was scanned. It was observed that there were significant changes in the expression of these genes between breast cancer tissues and adjacent tissues (1097 cases, Fig. 1a). The upregulation of *IRF7* or *CD3E* is positively correlated to the patients’ prognosis (Fig. 1b). IRF7 is a well-known transcription factor that regulates the secretion of interferon (IFN) in response to viruses^20^, which implies that the activation of innate immunity by viruses may further amplify the anticancer immunity (Supplementary Fig. 1). For example, IFN-γ is able to promote the production of IL12 by DCs, which process is required for the effective PD1/PDL1 blockade based immunotherapy for tumor^21^. The preparation of siRNA@HPVP is diagrammed in Fig. 1c. Three different siRNA sequences were designed and synthesized (denoted as siRNA1, siRNA2, and siRNA3). The *Cd274* knockdown effect of siRNA on 4T1 cells was evaluated by the real-time PCR analysis (Fig. 1d). According to the results, siRNA2 exhibited a superior knockdown effect than both siRNA3 and siRNA1. Although siRNA@HPVP was internalized by antigen-presenting cells (APCs), for these difficult-to-transfect cells, siRNA treatment did not effectively inhibit the expression of *Cd274* gene. After loading siRNA on HPVP, we measured the binding affinity between siRNA and HPVP with gel-electrophoresis assay^22^. It was found that the nucleic acids were effectively combined on HPVP with the mass ratio of 0.8:1 (siRNA:HPVP) (Fig. 1e). To ensure an adequate binding, an siRNA to HPVP ratio of 0.5:1 was used for material preparation.

From the protein structure analysis, both the hydrophobic effect and electrostatic interaction were estimated to be the driving force for the assembly of siRNA and HPV16 L1 protein (Supplementary Fig. 2a, b). The morphological structure of siRNA@HPVP was observed through the cryo-transmission electron microscope (cryo-TEM). siRNA@HPVP formed a uniform nanostructure with a diameter of about 60 nm (Fig. 1f). The protective effect of HPVP on siRNA was also studied by treating siRNA@HPVP with RNase. As shown in Supplementary Fig. 2c, naked siRNA was completely degraded by 5 mU of RNase. In contrast, HPVP protected siRNA from enzymolysis. Even after 2 months of storage at −20 °C, siRNA encapsulated in HPVP still remained. In addition, HPVP itself exhibited negligible cytotoxicity against the cells (Supplementary Fig. 2d).

To further demonstrate the internalization of HPVP by 4T1 cells, TEM was used. The clear viral structure was found in lysosomes of 4T1 cells (Supplementary Fig. 3a). In order to verify whether the interaction between HPVP and α_6_ integrin would enhance the endocytosis of HPVP, wide-type, and *Itga6*-knockout 4T1 (4T1^*Itga6*-^) cells were chosen and respectively treated with rhodamine B labeled HPVP. The endocytosis of HPVP was monitored at different time points. According to the results, the much higher HPVP uptake in 4T1 cells demonstrated that the endocytosis might be promoted by the interaction between HPVP and α_6_ integrin (Fig. 1g). Co-localization of HPVP and α_6_ integrin expressed on the 4T1 cells was also observed with the confocal laser scanning microscopy. The high Mander’s overlap co-localization of 0.93 confirmed that there existed interaction between HPVP and α6 integrin (Supplementary Fig. 3b).

### In vivo immunogenic effect of siRNA@HPVP

**Fig. 2:**
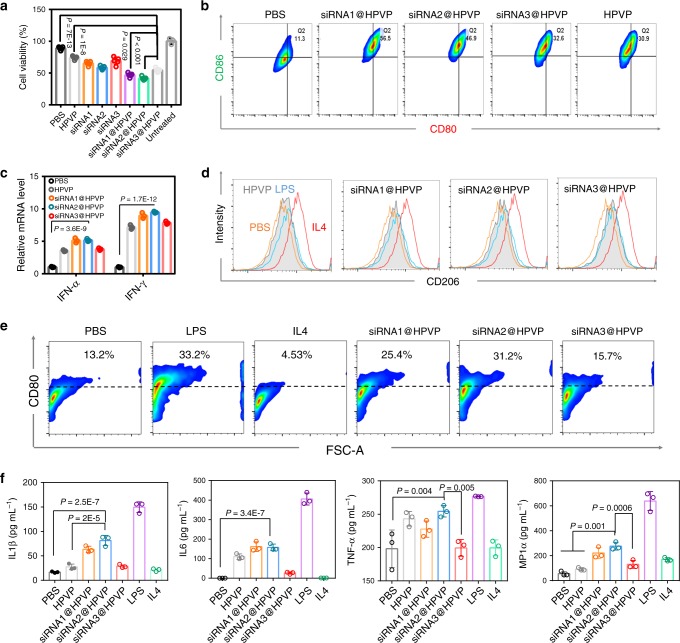
In vitro APCs activation. **a** In vitro cytotoxicity of splenocytes towards 4T1 cells (with the effector/target ratio of 10:1). Splenocytes were pretreated with different samples (PBS, HPVP, siRNA1, siRNA2, siRNA3, siRNA1@HPVP, siRNA2@HPVP, or siRNA3@HPVP) for 24 h before the cytotoxicity assay. Five biological replicates are shown. **b** FACS data of the mature DCs (CD80^+^CD86^+^) gating on CD11c^+^ cells after co-incubated with different siRNA@HPVP formats (siRNA1@HPVP, siRNA2@HPVP, or siRNA3@HPVP), HPVP or fresh culture medium for 24 h. A representative image of three biological replicates is shown. **c** Transcript abundance of IFN-α and IFN-γ in DCs after different treatments. Three biological replicates are shown. **d**, **e** FACS data to exhibit the phenotype and activation proportion of macrophages (CD11b^+^CD80^+^) 6 h after different treatments. M1 macrophages (CD11b^+^CD206^−^) were induced by lipopolysaccharide (100 ng mL^−1^). M2 macrophages (CD11b^+^CD206^+^) were induced by IL4 (20 ng mL^−1^). A representative image of three biological replicates is shown. **f** Cytokine levels in the culture medium of RAW 264.7 macrophages after different treatments. The concentration of HPVP for all in vitro immune stimulate assays was 20 mg L^−1^. Three biological replicates are shown. Statistical significance was calculated via one-way ANOVA with Tukey post-hoc analysis (**a**, **c**, **f**). Data are presented as mean values ± SD.

HPVP loading with siRNA1, siRNA2, and siRNA3 (denoted as siRNA1@HPVP, siRNA2@HPVP, and siRNA3@HPVP, respectively) were prepared. The immune-stimulating ability of three complexes was analyzed. First, the cytotoxicity effect of splenic cells towards 4T1 cells was studied with the lactate dehydrogenase (LDH) assay. As expected, the tumor cells were more vulnerable to splenic cells that pretreated with both siRNA1@HPVP and siRNA2@HPVP (Fig. 2a). In contrast, HPVP, siRNA3@HPVP, and siRNA3 oligonucleotides only slightly improved the immunotoxicity of splenic cells when compared with the PBS group.

APCs are the protagonist for inducing innate immunity^23^. Here, the influence of three siRNA@HPVP formats on APCs was studied. First, the influence of different treatments on dendritic cells (DCs) was analyzed with flow cytometry (FACS). Both HPVP and three kinds of siRNA@HPVP obviously promoted the maturation of DCs (Fig. 2b and Supplementary Fig. 3d). Among them, siRNA1@HPVP and siRNA2@HPVP exhibited better effect than siRNA3@HPVP. The maturation process of DCs induced by viruses is always accompanied by the activation of IFN signaling. So, we analyzed the expression levels of IFN-α and IFN-γ in DCs after different treatments (Fig. 2c). When compared with the PBS group, a 5.6-fold increase of *Ifna1* and 7.78-fold increase of *Ifng1* transcription were observed in siRNA2@HPVP-treated DCs. The same trend was also observed in siRNA1@HPVP-treated DCs. Meanwhile, the ELISA analysis showed that siRNA1@HPVP- and siRNA2@HPVP-treated DCs secreted the highest amount of IFN-α and IFN-γ (Supplementary Fig. 3e, f).

Besides, the effect of three siRNA@HPVP formats on macrophages polarization was also tested. After co-cultured with different samples, Raw 267.4 cells (murine macrophages) were analyzed with FACS (Fig. 2d). It was found that both siRNA@HPVP and HPVP skewed macrophages toward the M1 phenotype (CD11b^+^CD206^−^). Moreover, both siRNA1@HPVP and siRNA2@HPVP exhibited a better effect for inducing the activation of macrophages (CD11b^+^CD80^+^) than siRNA3@HPVP or HPVP (Fig. 2e). Besides, both HPVP and three siRNA@HPVP formats induced the secretions of IL1β, IL6, TNF-α, and MIP1α (markers of M1 macrophages). According to the quantitative analysis, siRNA1@HPVP and siRNA2@HPVP also exhibited more obvious effects than HPVP or siRNA3@HPVP (Fig. 2f). The differences among the immune-stimulating effects of the three siRNA might be due to the immune-stimulating sequences existing in siRNA1 or siRNA2^24^. As shown in Supplementary Fig. 3g–i, naked siRNA1 and siRNA2 also showed greater immunogenicity. Considering the immune-stimulating ability and *Cd274* knockdown effect of siRNA2@HPVP, this combination was chosen for further investigation.

### In vivo immunogenic effect evaluation of siRNA2@HPVP

The biocompatibility of siRNA2@HPVP was evaluated on guinea pigs (Supplementary Fig. 4a). The body temperature and lung histamine were measured after different treatments (Supplementary Fig. 4b, c). Negligible changes in body temperature and histamine levels indicated the allergenicity of siRNA2@HPVP. From blood biochemical test, no obvious hepatic and renal toxicity was found in siRNA2@HPVP-treated guinea pigs (Supplementary Fig. 4d), which also proved the good biosafety of siRNA2@HPVP. In addition, the blood levels of anti-PEG and antiviral antibodies after the injection of HPVP or HPV16 L1 protein were measured. As shown in Supplementary Fig. 4e, PEGylated materials produced less antiviral antibodies after the injection. No visible productions of anti-PEG antibodies were observed after the injection of PEGylated materials. At the same time, we also found that PEGylation stabilized the tumor enrichment effect of the repeatedly injected materials (Supplementary Fig. 4f).

**Fig. 3:**
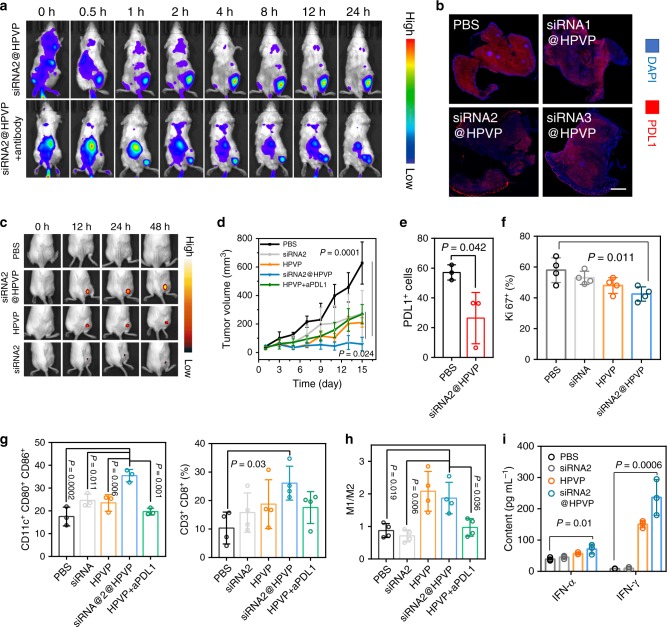
Tumor accumulation and anticancer ability of siRNA2@HPVP. **a** In vivo tumor-targeting capacity of HPVP on 4T1 tumor-bearing mice after *i.v*. injection. The blockade of α6 integrin with antibody reduced the tumor accumulation of HPVP. A representative image of three biological replicates is shown. **b** Ex vivo immunofluorescence images for the evaluation of PDL1 inhibition effect of siRNA1@HPVP, siRNA2@HPVP, and siRNA3@HPVP (Scale bar: 1 mm). A representative image of three biological replicates is shown. **c** Bioluminescence assay for the in situ measurement of innate immunity in 4T1 tumors after treated with siRNA2@HPVP. Tumor cells were transfected with a pTNF-α-promoter-luc plasmid for reporting the intratumoral TNF-α level. A representative image of three biological replicates is shown. **d** In vivo anticancer effect evaluation of siRNA2@HPVP and HPVP + aPDL1 on the subcutaneous murine breast tumor model. The tumor size was measured every other day. Five biological replicates are shown. **e** FACS analysis for measuring the expression of PDL1 protein in PBS- or siRNA2@HPVP-treated mice tumors. Three biological replicates are shown. **f** Quantitative analysis of Ki67^+^ tumor cells after different treatments. Four images per group were taken. **g** FACS data of the mature DCs (CD11c^+^CD80^+^CD86^+^) within lymph nodes (left) and tumor-infiltrating cytotoxic T cells (CD3^+^CD8^+^ T cells, right) after different treatments. Three biological replicates (right) and four biological replicates (left) are shown, respectively. **h** FACS analysis for measuring intratumoral M1/M2 macrophages ratio after different treatments. CD11b^+^CD206^+^ cells were defined as M2 macrophages, while CD11b^+^CD206^−^ cells were defined as M1 macrophages. Four biological replicates are shown. **i** IFN levels in the serum from the mice 12 h after different treatments. Three biological replicates are shown. Statistical significance was calculated via one-way ANOVA with a Tukey post-hoc test (**d**–**i**). Data are presented as mean values ± SD.

The tumor accumulation ability of HPVP was studied in 4T1 tumor-bearing mice. A noticeable tumor accumulation was observed 4 h after the intravenous injection with HPVP (Fig. 3a). Even 24 h after the injection, exceptionally intensive fluorescence was still visible in the tumor region. However, the tumor-targeting ability of HPVP was weakened after the blockade of α6 integrin with its antibody, suggesting that the tumor-targeting ability of HPVP relied on its biological interaction with α6 integrin expressed on the tumor cells. Twenty-four hours after injection, the mice were sacrificed to strip the tumor-draining lymph nodes, and the HPVP accumulation was also recorded. Attributed to the inhibition of tumor retention, more HPVP entered the lymph node (Supplementary Fig. 5).

Subsequently, the PDL1 inhibition capacity of siRNA1@HPVP, siRNA2@HPVP, and siRNA3@HPVP was also evaluated in vivo. According to the results of immunofluorescent staining analysis, siRNA1@HPVP, siRNA2@HPVP, and siRNA3@HPVP exhibited similar inhibition effect towards the expression of PDL1 in vivo (Fig. 3b). In contrast, a negligible PDL1 inhibition effect was found in the mice with HPVP injection, implying the PDL1 inhibition capacity of siRNA2@HPVP depended on the presence of siRNA. To visualize the immune responses, pTNF-α-promoter-luc^25^, a plasmid with TNF-α promoter-fused luciferase reporter gene, was designed and transfected into 4T1 tumors (4T1^pT^ tumor, Supplementary Fig. 5). 4T1^pT^ tumor-bearing mice with tumor volume around 200 mm^3^ were received with different treatments by *i.v*. injection. Progressively increased bioluminescence signaling was observed in the tumors of the siRNA2@HPVP group. The bioluminescence intensity reached its maximum intensity at 24 h after the injection. (Fig. 3c). The highest TNF-α level in tumor tissues of the siRNA2@HPVP group demonstrated the superior immunogenic effect of siRNA2@HPVP than HPVP and siRNA2.

After that, the anticancer effect of siRNA2@HPVP was investigated on subcutaneous 4T1 tumor-bearing mice. Mice were respectively administrated with siRNA2 (2.5 mg kg^−1^), HPVP (5 mg kg^−1^), and siRNA2@HPVP (7.5 mg kg^−1^) through *i.v*. injection, and the mice receiving PBS treatment were set as control. At day 15, siRNA2@HPVP exhibited a tumor inhibition rate of 83.5%, while monotherapy with HPVP or siRNA2 showed an inhibition rate of around 60% (Fig. 3d). FACS analysis also revealed that siRNA2@HPVP effectively decreased PDL1 expression in tumors (Fig. 3e). Besides, through the immunofluorescence staining, the number of Ki67^+^ cells (*P* = 0.011) significantly decreased in tumor samples from siRNA2@HPVP-treated mice, as compared with other groups (Fig. 3f). However, there was no statistical difference in the expression of Ki67, along with the groups treated by siRNA2, HPVP, and PBS. Taken together, the combination of innate immunity activation and PDL1 blockade exhibited excellent cooperative effect for anticancer therapy than any monotherapy. Interestingly, the direct use of aPDL1 + HPVP also slightly inhibited tumor growth. Although aPDL1 + HPVP was not as effective as siRNA2@HPVP, the combination of aPDL1 + HPVP might provide a simpler path to the clinic after further optimization.

Then, the in vivo immune-stimulating effect of siRNA2@HPVP was evaluated. Three days after the treatments, the tumor-draining lymph nodes of mice were harvested to evaluate the maturation states of DCs with FACS. siRNA2@HPVP treatment significantly (*P* = 0.001) promoted the maturation of DCs from 16.8 (PBS group) to 35.4%, which was around 1.5-fold more efficient than that of siRNA- or HPVP-treated group (Fig. 3g). Two weeks after the injection, tumor tissues were collected to analyze the tumor-infiltrating immune cells (Fig. 3g). The proportion of cytotoxic T cells (CD3^+^CD8^+^) in tumor tissues of mice treated with siRNA2@HPVP (31.5%) was higher than that of the groups treated with siRNA (12.6%) and HPVP (16.4%). Tumor-infiltrating macrophages were also analyzed by FACS. The ratio of M1 macrophages to M2 macrophages in the tumor site increased markedly (from 0.7 to 1.8) after both HPVP and siRNA2@HPVP treatments (Fig. 3h). Meanwhile, the levels of IFN-γ and IFN-α (mainly secreted by mature DCs) in the plasma were measured 12 h after the injection (Fig. 3i). As expected, high levels of IFN-γ and IFN-α were found in HPVP and siRNA2@HPVP groups. As expected, siRNA2@HPVP was more effective than HPVP for inducing IFN-γ and IFN-α secretion, which was attributed to the DCs maturation promoted by siRNA2. These results demonstrated that siRNA2@HPVP not only induced the innate immune defense but also activated a systematic anticancer cellular immunity.

### Transcriptomic analysis of the anticancer mechanism

**Fig. 4:**
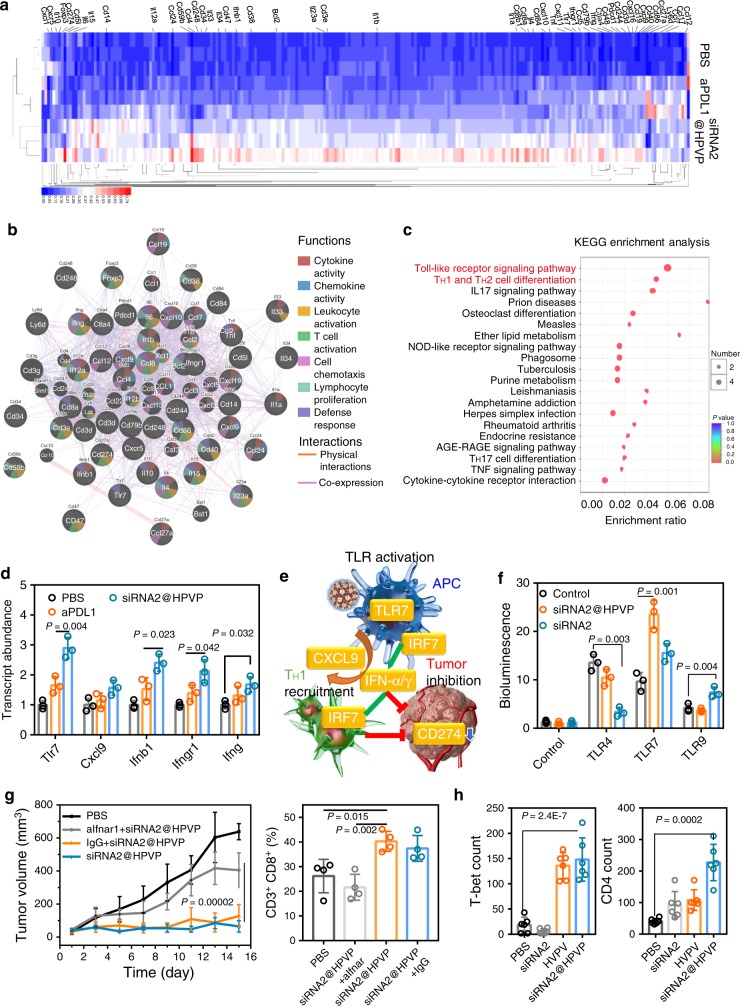
Activation mechanism of innate immunity. **a** Identification of differentially expressed genes in 4T1 tumors 3 days after intratumoral injection with siRNA2@HPVP or aPDL1. Differential genes in the GO term of the immune process were labeled. Three biological replicates are shown. **b** GeneMANIA for predicting gene interactions between differential genes in the GO term of the immune process. **c** KEGG enrichment analysis of the pathways involved in the biological effect induced by siRNA2@HPVP treatment. **d** PCR analysis of the transcript abundance of *Tlr7*, *Cxcl9*, *Infb1*, *Ifngr1*, and *Ifng* genes in 4T1 tumors after different treatments. Three biological replicates are shown. **e** Schematic diagram of the possible mechanism for siRNA2@HPVP to activate the innate immunity. siRNA2@HPVP activates APCs via the TLR7-mediate pathway, and then recruits T_H_1 cells for anticancer effect via their inherent immunogenicity and IFNs pathway. **f** HEK-293 cells transiently expressing TLR homodimers and ELAM-Luc reporter plasmid proved that the immune response mediated by siRNA2@HPVP is TLR7-dependent. Three biological replicates are shown. **g** Blockade of type I IFN pathway with anti-IFNAR1 antibody (aifnar, 200 μg per mice, *i.p*.) suppressed the therapeutic effect of siRNA2@HPVP (7.5 mg kg^−1^) treatment. Tumor size was recorded every other day (left, four biological replicates are shown). The proportion of tumor-infiltrating CD8^+^ T lymphocytes reduced when the IFN pathway was blocked (right, four biological replicates are shown). **h** Quantitative analysis of T-bet (left) and CD4 (right) expressions in 4T1 tumors through the immunofluorescence staining after different treatments. Tumor tissues were obtained 15 days after different treatments. Six images per group were taken. Statistical significance was calculated with two-tailed Student’s *t* test (**a**) one-way ANOVA with Tukey post-hoc analysis (**d**, **f**, **g**, **h**). Data are presented as mean values ± SD.

Three days after the peritumoral injection of siRNA2@HPVP, 4T1 tumor tissues were collected for transcriptomics analysis. A total of 14,679 genes were identified. Compared with the PBS group, 390 differential genes were found under a threshold with absolute fold changes >1.5 and *P* values < 0.05 (Fig. 4a and Supplementary Fig. 6). Genes associated with immune processes were sorted out (marked in Fig. 4a). Through the Gene Ontology (GO) analysis, most of the differential genes were found to enrich in the categories of defense response to virus infection (Supplementary Fig. 6). The more significant upregulation of the immune-related genes in the siRNA2@HPVP group than the other two groups demonstrated the remarkable immune-stimulating ability of siRNA2@HPVP over the aPDL1 treatment. Furthermore, GeneMANIA, a multiple association network integration algorithm for predicting gene interactions, was performed^26^. Most of these genes were corresponded to co-expression, following by physical interactions (Fig. 4b). As expected, a mass of genes associated with cytokine activity (31 of 121) and chemokine activity (16 of 30) was activated. Besides, changes of a gene cluster associated with T-cell activation (24 of 201), proliferation (21 of 161), and defense response of microbes (21 of 228), were also noticed. These results comprehensively demonstrated the immune activation ability of siRNA2@HPVP. Then, the Kyoto Encyclopedia of Genes and Genomes (KEGG) analysis was conducted to determine the detailed immune activation pathways mediated by siRNA2@HPVP treatment. From the KEGG pathway analysis, the most significant differences between the PBS and siRNA2@HPVP groups were found in the Toll-like receptor (TLR) signaling pathway (*P* = 0.0001) and T_H_1 and T_H_2 cell differentiation (*P* = 0.0011) pathways (Fig. 4c). For further verification, PCR analysis was performed to analyze the key genes of these two pathways. The expression levels of *Tlr7*, *Cxcl9*, *Ifnb1*, *Ifngr1*, and *Ifng* genes increased significantly after the siRNA2@HPVP treatment (Fig. 4d). From the above transcriptome analysis, we could propose the possible mechanism of siRNA2@HPVP for activating host immune response (Fig. 4e). In APCs such as DCs, siRNA2@HPVP activates the endosomal TLR signaling to promote antigen cross-presentation. In addition, siRNA2@HPVP triggered IFN-α/γ productions through the IRF7 pathway and recruited T-helper 1 (T_H_1) cells to regulate anticancer immunity^27^. Meanwhile, the knockdown of the *Cd274* gene with the corresponding siRNA further enhanced the anticancer immune response via preventing T cells from exhaustion.

To verify this speculation, HEK-293 cells transiently transfected with TLR homodimer and endothelial leukocyte adhesion molecule-luciferase (ELAM-Luc) reporter plasmid^28^ were stimulated by siRNA2@HPVP and siRNA, respectively. TLR7 and ELAM-Luc co-expressed HEK-293 cells displayed obvious responses toward both siRNA2@HPVP and siRNA (Fig. 4f). The results demonstrated that TLR7 played a major role in siRNA2@HPVP-mediated APCs activation. The inhibition of TLRs remarkably hindered the activation of APCs by siRNA2@HPVP at the cellular level.

To confirm the significant role of IFN signaling in the therapeutic mechanism of siRNA2@HPVP, an in vivo blocking experiment was conducted using an anti-IFNAR1 antibody. 4T1 tumor-bearing mice were randomly grouped and treated with PBS, siRNA2@HPVP plus IgG, siRNA2@HPVP plus anti-IFNAR1 antibody, and siRNA2@HPVP, respectively. As expected, the blockade of the IFN-α-mediated biological effect dramatically impaired the tumor inhibition effect of siRNA2@HPVP for 50% (Fig. 4g). Besides, significantly decreased tumor-infiltrating T cells were also observed after the antibody blockade. Similarly, siRNA@HPVP exhibited no obvious antitumor effect in immune-deficient NOD/SCID mice (Supplementary Fig. 7d). Moreover, a decrease in the therapeutic effect of siRNA2@HPVP was observed in the 4T1^*Itga6-*^ tumor-bearing mice. This result proved that the α6 integrin-mediated targeting was an important reason for the satisfactory therapeutic effect of siRNA2@HPVP (Supplementary Fig. 7e).

To explore the recruitment of T_H_1 cells (CD4^+^T-bet^+^), the immunofluorescent staining assay of tumor tissues was performed 3 days after the peritumoral injection of siRNA2@HPVP (Supplementary Fig. 8). An 8-fold increase of T-bet level, together with a 5.58-fold increase of CD4 level was observed in tumor tissues of siRNA2@HPVP-treated mice, as compared with that of the PBS group (Fig. 4h). This result was also in coincidence with our previous speculation.

### Anticancer effect in resectable and unresectable tumors

**Fig. 5:**
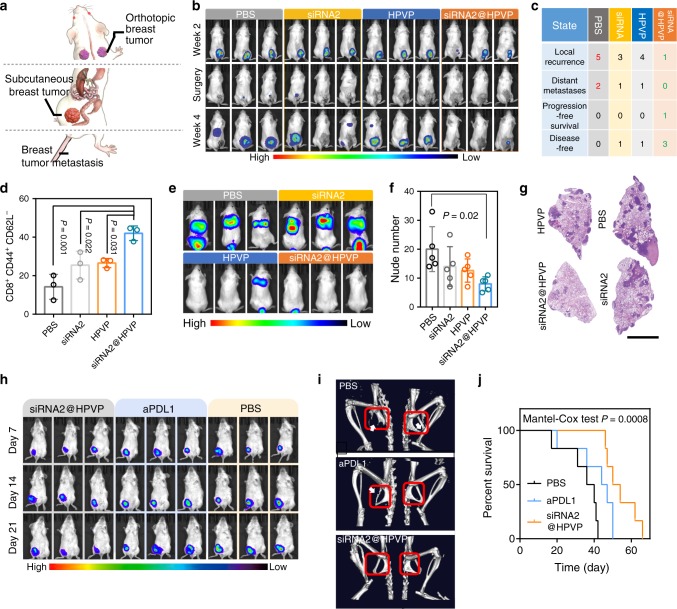
Therapeutic effect in resectable and unresectable tumor models. **a** Different tumor models established in this study for evaluating the anticancer effect of siRNA2@HPVP treatment. **b** In vivo bioluminescence images to track the recurrence and metastasis of 4T1 tumors after the resection of the primary tumor. The resection operation was conducted 15 days after different treatments. Three representative images of five biological replicates are shown. **c** The data of the lesion condition in the resectable murine breast tumor model after the resection of primary tumor. s@H represents siRNA2@HPVP. **d** The proportion of T_EM_ cells (CD8^+^CD44^+^CD62L^−^) in spleen tissues 40 days after different treatments. The primary tumor was removed by surgery at day 15. Three biological replicates are shown. **e** In vivo bioluminescence images to exhibit the anti-metastasis effect of various treatments. The metastatic breast tumor model was constructed by intravenously injecting BABL/C mice with 4T1^luc^ cells. The treatments were carried out 24 h after the tumor cells injection. Three representative images of five biological replicates are shown. **f**, **g** H&E staining and quantitative counting of metastatic nodes in lung tissues 21 days after the *i.v*. injection of 4T1 cells (Scale bar: 5 mm). Five images per group were taken. **h** In vivo bioluminescence images of orthotopic breast tumor-bearing mice after different treatments. In this assay, aPDL1 treatment as a clinical treatment for the unresectable tumor was also conducted. Three representative images of six biological replicates are shown. **i** Representative μ-CT images for the visualization of bone metastasis of orthotopic breast tumor after various treatments. Sites of osteolysis are circled. **j** Survival curve of orthotopic breast tumor-bearing mice with various treatments. Six biological replicates are shown. Statistical significance was calculated via one-way ANOVA with Tukey post-hoc test (**d**, **f**) and Logrank test (two-sided) for trend (**j**). Data are presented as mean values ± SD.

The anticancer effect of siRNA2@HPVP was also tested on various murine tumor models (Fig. 5a). Clinically, tumor recurrence or metastasis after surgery is the main cause of the treatment failure, and seeking better treatments to overcome those problems is of great importance^29^. In this study, the inhibition effect of siRNA2@HPVP on local tumor recurrence was evaluated. One week after the subcutaneous inoculation of luciferase-transfected 4T1 (4T1^luc^) cells, mice were treated with various agents (PBS, siRNA, HPVP, and siRNA2@HPVP). Two weeks after the treatment, the tumors were removed by the surgery, and the tumor recurrence was monitored with the bioluminescence imaging. In the PBS group, the local tumor recurrence was observed in all mice, and two of them showed distant metastasis (Fig. 5b, c). Even in siRNA- and HPVP-treated groups, the local recurrence or distant metastasis was found in more than 50% of mice. In siRNA2@HPVP-treated mice, the local recurrence only occurred on one mouse, and no distant metastasis was found during the treatment. To explain this phenomenon, the proportion of effector memory T (T_EM_) cells (CD8^+^CD44^+^CD62L^−^) in the spleen was analyzed^30^. As expected, the highest level of splenic T_EM_ cells was found in the siRNA2@HPVP group (42%), which was approximately threefold higher than that of the PBS group (Fig. 5d). This result indicated the inhibition of recurrence by siRNA2@HPVP treatment was mainly attributed to the activation of immunological memory effect.

Then, the anti-metastasis performance of siRNA2@HPVP was studied. The 4T1^luc^ cells were intravenously injected into the mice to simulate the circulating tumor cells. Two weeks after the treatment, massive pulmonary metastases were found in PBS and siRNA groups. Very limited tumor metastases were found in siRNA2@HPVP-treated mice, and this might benefit from the robust immune effect by HPVP (Fig. 5e). At the end of the therapy, mice were sacrificed, and their pulmonary metastatic tumors were counted under a stereomicroscope. As compared with the PBS group, siRNA2@HPVP treatment inhibited 60% of macroscopical metastasis in the lung (Fig. 5f). Fewer metastases in siRNA2@HPVP-treated mice were found in H&E staining images (Fig. 5g).

Developing therapies to prolong patients’ survival is the main task for treating unresectable advanced tumors^3^. Atezolizumab, the antibody of PDL1, has been approved by the US FDA for the treatment of patients with solid tumors^31^. In this study, the therapeutic effects of siRNA2@HPVP and aPDL1 were compared on the unresectable murine orthotopic breast tumor model. At the initial stage (~3 weeks) of siRNA2@HPVP treatment, the average volume of mice tumors was nearly unchanged. aPDL1 treatment also slightly inhibited the tumor growth, but less effective than the siRNA2@HPVP treatment (Fig. 5h). Four weeks after different treatments, μ-CT imaging was performed to visualize spontaneous metastasis/invasion from the mammary gland to the bone. Osteolytic bone metastases/invasion were clearly observed in the pubis and ischium of PBS-treated mice (Fig. 5i)^32^. In aPDL1-treated mice, the slight lysis of pubis was observed. As a contrast, no obvious osteolysis was found in siRNA2@HPVP-treated mice. In the 34th day of therapy, 50% of mice in the PBS group died, and 66% of mice of the aPDL1 group were still alive. Whereas, none of the mice in the siRNA2@HPVP group died during this period (Fig. 5j).

The therapeutic effect of siRNA2@HPVP was also tested on a murine orthotopic colon tumor model. The tumor growth was monitored by measuring its bioluminescence intensity. siRNA2@HPVP treatment effectively inhibited the growth of primary tumors and prevented tumors from invasion (Supplementary Fig. 9a). Four weeks later, mice were sacrificed, and their intestinal tracts were stained with methylene blue to observe the invasive tumors. The least tumor invasion was found in the siRNA2@HPVP group in comparison with the other two groups (Supplementary Fig. 9b, c). From these results, siRNA2@HPVP treatment might be applicable for cancer types other than TNBC.

### The response rate of siRNA2@HPVP treatment

**Fig. 6:**
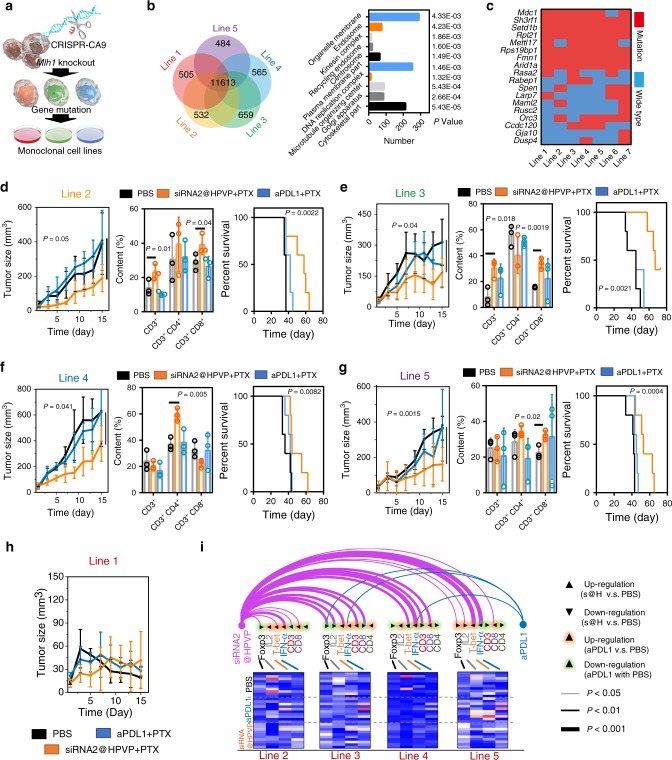
The therapeutic response rate of siRNA2@HPVP treatment. **a** Schematic illustration of the CRISPR-Cas9-mediated *Mlh1* knockout for large-scale gene mutations. Seven monoclonal 4T1 cell lines with different gene mutations were purified. Five cell lines were respectively inoculated on the mice to evaluate the immune response rate of siRNA2@HPVP treatment. **b** SNP (left) and Indel (right) analyses of the obtained seven mutant cell lines. The union graph of total mutation among five mutant cell lines was constructed to evaluate the connection between the five cell lines. **c** AS analysis of the mutation of the cell lines after *Mlh1* knockout. **d**–**h** Tumor growth curves (left, five biological replicates are shown), tumor-infiltrating T cells proportions (middle, three biological replicates are shown) and survival curves (right, five biological replicates are shown) of five distinct breast tumor models with aPDL1 plus PTX (1 mg kg^−1^ aPDL1 and 10 mg kg^−1^ PTX) or siRNA2@HPVP plus PTX (7.5 mg kg^−1^ siRNA2@HPVP and 10 mg kg^−1^ PTX) treatment by *i.v*. injection (*n* = 5 per group). Three tumors per group were collected for flow cytometry analysis. **i** Arc plot and heat map for illustrating the intratumoral immune indexes (CD3, CD4, CD8, Foxp3, IL2, T-bet, and IFN-γ) in each individual. Two locations were taken for each tumor. Statistical significance was calculated via two-tailed Student’s *t* test (**b**, **d**–**g**, **i**) and Logrank test (two-sided) for trend (**d**–**g**). Data are presented as mean values ± SD.

The low response rate in the clinic is a great obstacle to the development of ICB therapy. Therefore, we investigated the response rate of siRNA2@HPVP treatment. Previously, atezolizumab (anti-PDL1 antibody) plus paclitaxel (PTX) treatment has been done in a phase III clinical trial for extending progression-free survival and overall survival^33^. Thus, we compared the therapeutic effect between siRNA2@HPVP plus PTX and aPDL1 plus PTX on five breast cancer models with different antigen loads. To construct the mutant breast cancer models, the MutL homolog 1 (*Mlh1*) gene in 4T1 cells was genetically inactivated by CRISPR-Cas9 technology, which would lead to dynamic mutation^34^. Then, the monoclonal culture of cells was performed, and stable monoclonal 4T1 cell lines with varied mutations (4T1^mut^) were obtained (Fig. 6a). Single nucleotide polymorphisms (SNP) and insertion-deletion (Indel) analyses were performed on these 4T1^mut^ cell lines to identify the mutant genes. About 500 distinct mutations were found in each cell line (Supplementary Data 1). Then, GO analysis was performed to analyze the functions of the mutant genes. According to the results, these genes were found to be expressed in nearly all organelles of tumor cells (Fig. 6b). Next, the alternative splicing (AS) analysis for these cell lines was also obtained (Fig. 6c). Through the result of AS analysis, a series of large mutations were discovered, which also implied the successful mutagenesis.

The subcutaneous tumor models of these 4T1^mut^ cell lines were established to simulate the patient tumors with individual differences. The therapeutic effect of siRNA2@HPVP + PTX was compared with the clinical aPDL1 plus PTX treatment. aPDL1 plus PTX treatment showed significant inhibition only in 4T1^mut-3^ tumors (Fig. 6d–h). In contrast, tumor inhibition rates of over 40% were observed in 4T1^mut-2^, 4T1^mut-3^, 4T1^mut-4^, and 4T1^mut-5^ tumors with siRNA2@HPVP plus PTX treatment. After the treatment, the tumor-infiltrating lymphocytes were analyzed to evaluate the anticancer immunity of different treatments further (Fig. 6d–g). As expected, the siRNA2@HPVP + PTX treatment promoted the infiltration of T-helper cells (CD3^+^CD4^+^) in 4T1^mut-2^, 4T1^mut-4^, and 4T1^mut-5^ tumors. Increased cytotoxic T cells proportion (CD3^+^CD8^+^) was also observed in 4T1^mut-2^, 4T1^mut-3^, and 4T1^mut-5^ tumors, thus leading to an efficient anticancer effect. In addition, siRNA2@HPVP significantly prolonged the survival of mice in four different tumor models (Fig. 6d–g). Unexpectedly, tumors of 4T1^mut-1^ tumor-bearing mice disappeared after the treatment (Fig. 6h). This might be attributed to the high immunogenicity of 4T1^mut-1^ cells. Later, immune-responses-associated indexes (CD3, CD4, CD8, Foxp3, IL2, T-bet, and IFN-α) were analyzed by the on-chip immunofluorescence detection (Supplementary Fig. 10). These data are presented with the Act plot in Fig. 6i^35^. When compared with the PBS group, the upregulation of the T-bet and IFN-α expression, together with the downregulation of Foxp3 level, were observed in the siRNA2@HPVP plus PTX group compared with the PBS group. The contribution of these factors towards the anticancer effect of siRNA2@HPVP plus PTX treatment was analyzed with the logistic regression test. IFN-α, the core factor of antiviral immunity, showed the highest B factor of 18.513. Together, the antiviral immunity induced by siRNA2@HPVP is efficient in improving the response rate of PDL1 blockade immunotherapy.

## Discussion

To sum up, the HPV pseudovirus nanoparticles loaded with the PDL1 blockade siRNA (siRNA@HPVP) were obtained for effective anticancer immunotherapy with a high response rate. siRNA@HPVP exhibited good tumor accumulation ability via the active targeting effect mediated by the vaccine and α6 integrin on tumor cells. More importantly, HPVP effectively activated the innate immune to cooperate with ICB therapy of tumors. The preoperative siRNA2@HPVP treatment was proved to reduce the postoperative recurrence and distant metastasis in resectable breast cancer. In an unresectable tumor model, compared with the clinical-used regimen, siRNA2@HPVP also extended the progression-free survival and overall survival. Moreover, when combined with the chemotherapy of PTX, siRNA2@HPVP exhibited satisfactory and stable therapeutic response rate on a series of heterogenetic tumor models, superior to the treatment in phase III clinical trials (aPDL1 plus PTX). This meeting between the clinically used vaccine and ICB might provide methods for updating current cancer therapy.

Interestingly, it was found that the combination of PDL1 antibody with HPVP also improved its therapeutic effect. Whereas, in the mouse subcutaneous tumor model, it was demonstrated that siRNA@HPVP had a better tumor-suppressive effect than PDL1 antibody + HPVP. In siRNA-based therapy, HPVP encapsulated and delivered siRNA to tumor sites. In contrast, due to the large molecular weight of the PDL1 antibody, it could not be encapsulated by HPVP. Rapid elimination and off-targeting of antibodies were inevitable^36,37^. Therefore, the siRNA could more accurately silence the *Cd274* gene of tumors. In this study, siRNA has an advantage over antibodies, because siRNA benefited from both targeting and immune effect of HPVP.

However, from a practical point of view, the combination of HPVP and antibody has better translational potential. Both HPV vaccine and PD1/PDL1 antibodies have been approved for clinical applications. If the combination of HPVP and antibodies is effective in large animal experiments and clinical trials, there will be a simple path to clinical practice. Recently, givosiran and patisiran, two siRNA-based drugs, have been approved for clinical uses^38^. These successes also demonstrate the great potential of siRNA in cancer immunotherapy. Although the application of siRNA therapy is still in its infancy, we are still looking forward to the further clinical translation of siRNA@HPVP.

## Methods

### Materials

Recombinant HPV16 L1 protein was purchased from Biodragon Immunotechnologies. N-hydroxylsuccinimide functionalized polyethylene glycol2000 (PEG_2000_-NHS) was purchased from Ponsure Biotech, Inc. Anti-mouse PDL1 (cat. no. BE0101, Clone, 10 F.9G2) and anti-mouse IFNAR1 (cat. no. BE0241, Clone, MAR1-5A3) were supplied by Bioxcell. Anti-mouse CD16/32 (cat.no. 101329, Clone, 93), FITC-anti-mouse CD3 (Cat.no. 100203, Clone, 17A2), PE-anti-mouse CD4 (cat.no. 100407, Clone, GK1.5), APC-anti-mouse CD8a (cat.no. 100711, Clone, 53-6.7), FITC-anti-mouse CD8a (cat.no. 100705, Clone, 53-6.7), PE-anti-mouse CD11c (cat.no. 117307, Clone, N418), FITC-anti-mouse CD80 (cat.no. 104705, Clone, 16-10A1), APC-anti-mouse CD86 (cat.no. 105011, Clone, GL-1), PE-anti-mouse CD11b (cat.no. 101207, Clone, M1/70), APC-anti-mouse CD206 (cat.no. 141707, Clone, C068C2), APC-anti-mouse CD44 (cat.no. 103011, Clone, IM7) and PE-anti-mouse CD62L (cat.no. 104407, Clone, MEL-14) antibodies were all purchased from BioLegend. IFN-α polyclonal antibody (cat.no. bs-6304R, Clone, Polyclonal) was bought from the Bioss Guarantee. Anti-IFN-γ (cat.no. ab9657) was purchased from Abcam. D-luciferin potassium salt was bought from Shanghai Yeasen. HRP-labeled goat anti-mouse IgG (cat.no. A0216, Source, goat), puromycin, 4% paraformaldehyde solution, pTNF-α-luc plasmid, GM-CSF and IL4 were bought from Beyotime. Methylene blue, DTT, glutaraldehyde, glucose, cyanine5 NHS ester, and rhodamine B isothiocyanate were supplied by Aladdin. Hydroxychloroquine sulfate was purchased from Selleck. 1,2-Dioleoyl-sn-glycero-3-phosphoethanolamine (DOPE) and DC-cholesterol (DC-Chol) were bought from Sigma-Aldrich. Roswell Park Memorial Institute (RPMI) 1640 culture medium, Gibico®Opti-MEM medium, fetal bovine serum (FBS), penicillin–streptomycin and trypsin were purchased from Invitrogen. Pierce™. LDH cytotoxicity assay kit, lipofectamine™ 2000 and Lipofectamine™ 3000 transfection reagents were all purchased from Thermo Scientific. ELISA kits for IL1β (cat.no. CME0015), IL6 (cat.no. CME0006), TNF-α (cat.no. CME0004), and IFN-γ (cat.no. CME0003-10*96) were purchased from 4A Biotech Co., Ltd. ELISA kit for IFN-α (cat.no. SEKM-0149) was purchased from Beijing Solarbio Science & Technology Co., Ltd. ELISA kits for MIP1α (cat.no. SEA092Mu) and histamine (cat.no. CEA927Ge) were bought from USCN Business Co., Ltd.

### Cell lines

3T3 mouse embryonal fibroblasts, B16 murine melanoma cells, 4T1 murine breast tumor cells, CT26 murine colon tumor cells, Raw 267.4 murine macrophages, and HEK-293 cells were obtained from China Center for Type Culture Collection (CCTCC). 4T1^*Itga6*-^cells were obtained using the Cas/CRISPER technology. Bone marrow-derived dendritic cells (BMDCs) were extracted from the C57BL/6 mice with the following steps. First, both femur bones obtained from one mouse were sterilized in 70% ethanol on ice for 5 min. Then, the bones were transferred to the petri dish and immersed in 10 mL of RPMI 1640 culture medium, and the epiphesis of each bone was cut to expose the bone marrow. Next, the bone was held with sterile tweezers and inserted by the needle of one syringe containing 3 mL of medium, and the bone marrow was repeatedly washed with the medium. The obtained cell suspension was filtered through a 70-μm nylon cell strainer, and the cells were collected by centrifugation (1500 rmp, 5 min). Before use, BMDCs were stimulated with IL4 (10 ng mL^−1^) and GM-CSF (20 ng mL^−1^) in RPMI 1640 culture medium for 7 days.

### Software

All statistical analyses were performed on Origin (version 8.6), SPSS (version 22) or Excel 2016. Living image software (Version 4.5) was used to analyse bioluminescent and fluorescent images. Image J (Version 1.48h3) was used for fluorescence-image analysis. Transcriptomics data were analyzed online with I-Sanger Cloud Platform (https://cloud.majorbio.com). Gensys (Version1.6.9.0) was used to image the agarose gel. FlowJo (Version 4.5) was used for flow cytometry analysis. The gating strategy is listed in Supplementary Fig. 11.

### Preparation and characterization of siRNA@HPVP

Five milligrams of HPV16 L1 protein assembly was disassembled with 2 mM of DTT in PB buffer solution (pH 8.2, containing 0.166 M NaCl) for 1 h at 25 °C. Then, 50 μg of PEG_2000_-NHS and a certain amount of siRNA (with the mass ratio of HPV/siRNA  = 0.5, 1, 2, 4, 8, 10, and 15) were added to the mixed solution. After that, the obtained solution was dialyzed against PB buffer (containing 0.5 M NaCl) for 24 h to reassemble. The binding affinity of siRNA and HPV protein was evaluated via the gel-electrophoresis assay. The morphology structure of the siRNA@HPVP (with a mass ratio of HPV/siRNA = 0.5) was observed by the TEM (JEOL-2100) and Cryo-TEM (FEI Tecnai 20). The sample was negatively stained with 1% phosphotungstic acid before observation. For the agarose electrophoresis experiment, different samples containing 0.5 μg of siRNA were loaded in a 2.5% agarose gel, and 60 mV was applied for 30 min. 1 × TAE buffer was used as the running buffer.

### In vitro cell uptake

After seeded and cultured for 24 h in glass bottom dishes (1 × 10^5^ cells per dish), 4T1 cells were co-cultured with the HPVP (30 mg L^−1^ in RPMI 1640 culture medium) for 3 and 6 h, respectively. After being washed and collected, the cells were fixed with 2.5% glutaraldehyde for 24 h. The morphology structure of intracellular siRNA@HPVP was observed with TEM (HITACHI, HT7700). Next, the targeting ability of HPVP for α6 integrin was investigated by FACS. For this purpose, the HPVP was labeled with rhodamine B (HPVP-RB) following the steps below. HPVP (100 μg, 1 g L^−1^) was stirred with rhodamine B isothiocyanate (1 μg, 10 μg L^−1^) at room temperature. Twelve hours later, the purified HPVP-RB was collected via the cross-flow ultrafiltration system fitted with a GE-Osmonics GE series membrane (100 kDa). 4T1 cells and 4T1^*Itga6-*^ cells were seeded in the 6-well plates (1 × 10^5^ cells per well), respectively. After cultured for 24 h, the cells were treated with 1 mL of HPVP-RB nanoparticles (20 mg L^−1^). After the preset time points (6, 12, and 24 h), the cell supernatant was discarded and the cells were washed with PBS for three times. The endocytosis of HPVP was detected by FACS.

### In vitro immune stimulation assays

DCs (extracted from the bone marrow following the steps described above) or macrophages were seeded in six-well plates (1 × 10^6^ cells each well), and then co-cultured with PBS, siRNA1@HPVP, siRNA2@HPVP, siRNA3@HPVP, and HPVP (HPVP: 20 mg L^−1^, siRNA: 10 mg L^−1^, 24 h for DCs and 6 h for macrophages). Before detection, the supernatant was collected and the cells were washed with PBS for three times. For FACS analysis, DCs cells were blocked by anti-CD16/32 antibody and then stained with anti-mouse CD11c, anti-mouse CD80, and anti-mouse CD86 (mature DCs: CD11c^+^CD80^+^CD86^+^). Macrophages were blocked with anti-CD16/32 antibody and then stained with anti-mouse CD11b, anti-mouse CD80 and anti-mouse CD206 antibodies (M1 phenotype: CD11b^+^CD80^+^CD206^−^, M2 phenotype: CD11b^+^CD80^+^CD206^+^). The cytokines (IL1β, IL6, TNF-α, and IFN-γ) in supernatant of macrophages were analyzed by ELISA kits. In addition, the immune-stimulating effects of the three siRNA sequences were also explored on the macrophages. After the treatment with lipo-siRNA1, lipo-siRNA2, and lipo-siRNA3 for 24 h, the IL6 and IFN-γ levels in the supernatant of Raw 267.4 were detected by ELISA. What’ more, the activation marker (CD80) of macrophages was also analyzed by FACS.

### Tumor models

All animal studies were approved by the Institutional Animal Care and Use Committee (IACUC) of the Animal Experiment Center of Wuhan University (Wuhan, China). All mouse experimental procedures were carried out following the Regulations for the Administration of Affairs Concerning Experimental Animals approved by the State Council of People’s Republic of China. All animals were anesthetized by isoflurane and sacrificed by CO_2_-euthanasia. For humane reasons, animals were sacrificed when the solid tumor volume exceeded 2000 mm^3^. We constructed four different tumor models based on female Balb/c mice. 4T1^luc^ cells (1 × 10^6^ cells per mouse) were subcutaneously injected in the right flank of the mice to build the subcutaneous breast tumor model. The mice were intravenously injected with 4T1^luc^ cells (1 × 10^5^ cells per mouse) to simulate circulating tumor cells. Meanwhile, the orthotopic tumor models were also constructed. After sterilized with alcohol spray, the mice were taken to an aseptic environment. For orthotopic breast tumor model, the mammary fat pad was picked out and injected with 4T1^luc^ cells (1 × 10^6^ cells per mouse), and the wound was closed using Vetbond (3 M). In order to establish an orthotopic colon tumor model, the peritoneum was scissored to form a median incision at the lower ventral abdominal, and the cecum was exteriorized. CT26^luc^ cells (1 × 10^6^ cells per mouse) were injected to the cecal wall. After the return of cecum, the wound was closed using Vetbond (3 M).

### In vivo antitumor effect

When the subcutaneous 4T1 tumor was implanted for ~5 days, the mice were randomly divided into four groups (*n* = 5), and then received the following treatments by *i.v*. injection of PBS, liposome-encapsulated siRNA2, HPVP, HPVP plus aPDL1, and siRNA2@HPVP (equivalent to 5 mg kg^−1^ bodyweight of HPVP, 2.5 mg kg^−1^ bodyweight of siRNA, 1 mg kg^−1^ bodyweight of aPDL1), respectively. The day of the treatment was denoted as day 1. Then the tumor size and mice bodyweight were recorded every 2 days. The tumor volume (V_t_) was calculated as the following formula: V_t_ = L × W^2^/2, in which L and W, respectively, represent the longest and shortest lengths of the tumor. Meanwhile, at day 15, the tumor tissues of different groups were excised by surgery and fixed with the paraformaldehyde solution (4%) for immunohistochemical analysis. Besides, the intratumoral PDL1 expression levels of different groups were analyzed by FACS. The tumor metastasis and recurrence state of the mice were recorded 2 and 4 weeks after operation by the bioluminescence on the IVIS Spectrum. The antitumor effect of siRNA2@HPVP was also evaluated on the 4T1 tumor-bearing NOD/SCID mice and the 4T1^*Itga6*-^ tumor-bearing BABL/c mice models through the intravenous injection (with the dose of 5 mg kg^−1^ bodyweight of HPVP and 2.5 mg kg^−1^ bodyweight of siRNA). The tumor size was recorded every other day until day 15.

For anti-metastasis effect analysis, after 24 h of *i.v*. injection with 4T1^luc^ cells, the mice were also treated with PBS, siRNA2, HPVP, and siRNA2@HPVP (equivalent to 5 mg kg^−1^ bodyweight of HPVP and 2.5 mg kg^−1^ bodyweight of siRNA) via *i.v*. injection. Fourteen days after the treatment, the distant metastasis was monitored by the bioluminescence. After that, the mice were sacrificed and lung tissues were obtained for H&E staining.

The antitumor effect of siRNA2@HPVP on the orthotopic breast tumor model was also evaluated. After the inoculation of tumor cells for 5 days (denoted as day 1), the mice were respectively treated with PBS, aPDL1 (1 mg kg^−1^) and siRNA2@HPVP (7.5 mg kg^−1^). Treatments were performed once a week. The growth of tumor was monitored through the bioluminescence every week until day 21. At day 21, the bone metastasis state of the mice with different treatments was observed via μ-CT images on the Quantum GX micro CT imaging system (PerkinElmer). To explore the antitumor effect of siRNA2@HPVP against colon tumor, different treatments (PBS, 1 mg kg^−1^ aPDL1 and 7.5 mg kg^−1^ siRNA2@HPVP) were conducted 5 days after the tumor inoculation (day 1). The growth of tumor was monitored through the bioluminescence every week until day 21. At day 21, for the observation of tumor invasion state in the orthotopic colon tumor-bearing mice, the intestinal tracts of the mice were obtained and soaked into the paraformaldehyde solution (4%) for 2 h. Then, methylene blue solution (0.5%) was used to stain the intestinal tracts for 30 s. After being washed with PBS for several times, the intestinal tracts were observed on a stereomicroscope (Olympus). The tumors on the intestine were stained into blue by methylene blue. Besides, the intestinal tracts were stained with H&E. The colon tumor invasion nodules were counted through the H&E images.

### In vitro *Cd274* knockdown

Control siRNA sequence, siRNA1, siRNA2, and siRNA3 were respectively encapsulated with liposome (denoted as lipo-control sequence, lipo-siRNA1, lipo-siRNA2, and lipo-siRNA3) with the following steps. Diluting Lipofectamine 2000 (0.75 μL) with 25 μL of Gibico®Opti-MEM, and diluting 500 ng of siRNA with 25 μl of Gibico^®^Opti-MEM. Next, the diluted Lipofectamine 2000 and diluted siRNA were mixed and incubated for 5 min. After being seeded and cultured in six-well plate for 24 h, 4T1 cells were treated with blank medium, lipo-control sequence, lipo-siRNA1, lipo-siRNA2, and lipo-siRNA3 for 24 h, respectively. Raw 267.4 cells were treated with lipo-siRNA2 for 24 h. The *Cd274* knockdown effect was then measured with the real-time PCR. In detail, after being extracted from the treated cells, mRNA was subjected to the reverse transcription to obtain cDNA. The reverse transcription system contained 5 μg of RNA, 2 μL of Oligo (dT) 18 (10 μM), 4 μL of dNTP (2.5 mM), 4 μL of hiscript buffer (5×), 1 μL of hiscript reverse transcriptase, 0.5 μL of ribonuclease inhibitor, and ultrapure water with the total volume of 20 μL. After the sufficient mixing, the reaction was successively placed in 25 °C for 5 min, 50 °C for 15 min, 85 °C for 5 min, and 4 °C for 10 min. The obtained cDNA was diluted for ten times, and then quantified by the real-time fluorescence PCR analysis. The PCR reaction system contained 0.4 μL of forward primer (10 μM), 0.4 μL of reverse primer (10 μM), 10 μL of SYBR Green Master Mix, 0.4 μL of ROX reference dye 2 (50×), and 4.8 μL of ultrapure water. After the sufficient mixing, the reaction was successively placed in 50 °C for 2 min, 95 °C for 10 min, 95 °C for 0.5 min, 60 °C for 0.5 min, and then repeated for 40 times. Following that, the dissolution curve was drawn and the data was analyzed with the 2^−ΔΔCt^.

### Detection of antibodies to HPV protein and PEG

The naive mice about ~7 weeks were divided into three groups (*n* = 3) and then administrated with HPV protein (100 μg per mouse), HPVP (containing 100 μg of HPV protein), and PBS via intravenous injection, respectively. Three days after the injection, the serum samples of different groups were collected to analyze the antibody levels of HPV protein and PEG with ELISA. In detail, 96-well plate was coated with HPV and PEG solution (100 μL per well, 5 μg L^−1^) at 4 °C for 12 h. After that, the plate was washed with PBS (containing 0.05% Tween-20) for three times and the blocked with 1% BSA (200 μL each well) at room temperature for 2 h. After the appropriate dilution, samples (100 μL each well) were added to the well of the plate and incubated for another 12 h. Afterward, the horseradish peroxidase-conjugated goat anti-mouse immunoglobulin reagents specific for total mouse IgG was added to the plate (50 μL each well) following three times washing with PBS. After 2 h, the plate was washed with PBS for six times and incubated with TMB substrate (60 μL each well) for 30 min for color development. The reaction was then stopped by H_2_SO_4_ (2 N, 60 μL each well). Optical densities (OD) were recorded by the microplate reader (Thermo Scientific Multiskan Go).

### Cytotoxicity of siRNA2@HPVP

4T1 cells were seeded in a 96-well plate (5000 cells per well) and cultured for 24 h. Then, the fresh medium containing different concentration of siRNA2@HPVP nanoparticles were added to the plate and incubated for another 24 h. After that, with the addition of 3-[4,5-dimethylthiazol-2-yl]-2, 5-diphenyltetrazolium-bromide solution (20 μL each well, 5 mg mL^−1^) for 4 h, the OD was measured with the microplate reader. The cell viability was calculated following the formula: cell viability = 100% × (OD_(samples)_ − OD_(blank)_)/(OD_(control)_ − OD_(blank)_). The OD_(samples)_ and OD_(control)_, respectively, represent the absorbance of the sample-treated wells and culture medium-treated wells, while the OD_(blank)_ represent the absorbance of blank cells.

### Cytotoxic T lymphocyte activity

Splenocytes harvested from the Balb/c mice were stimulated with blank medium, HPVP, lipo-siRNA1, lipo-siRNA2, lipo-siRNA3, siRNA1@HPVP, siRNA2@HPVP, and siRNA3@HPVP, respectively. Then, the cells were washed three times with PBS and co-cultured with 4T1 cells at the ratio of 10:1 in a 96-well plate for 24 h. The suspension of each hole was collected and LDH leakage was detected via the nonradioactive cytotoxicity assay. The cell viability was calculated as follows: cell viability = ((experimental LDH release − effective cell LDH release)/(maximum LDH release − spontaneous LDH release)) × 100%.

### siRNA sequence

*Pdl1*-siRNA1 sense: CUACGGGCGUUUACUAUCATT; anti-sense: UGAUAGUAAACGCCCGUAGTT. *Pdl1*-siRNA2 sense: GAAGGGAAAUGCUGCCCUUTT; anti-sense: AAGGGCAGCAUUUCCCUUCTT; *Pdl1*-siRNA3 sense: GAGGAUAUUUGCUGGCAUUTT; anti-sense: AAUGCCAGCAAAUAUCCUCTT.

### Biosafety test

Dunkin-Hartley guinea pigs were exploited to evaluate the biocompatibility of siRNA2@HPVP. After the subcutaneous injection with siRNA2@HPVP (500 μg per mouse) or OVA (500 μg per mouse), the body temperature of guinea pigs (*n* = 3) was recorded every 10 min for 1 h. Three days after the injection, the blood samples of siRNA2@HPVP- and PBS -treated guinea pigs were collected for the blood biochemistry and blood routine analyses. The lung histamine concentration was measured with histamine ELISA kit.

### In vivo imaging

HPVP (500 μg, 1 mg mL^−1^) was labeled with cyanine5 (Cy5) by stirring with Cy5-NHS (5 μg, 10 μg mL^−1^) for 12 h. The Cy5 labeled HPVP was purified by the cross-flow ultrafiltration system fitted with a GE-Osmonics GE series membrane (100 kDa). When the tumor size reached around 100 mm^3^, the subcutaneous 4T1 tumor-bearing mice were divided into two groups. For one group, the mice were intraperitoneally injected with α6 integrin antibody (10 mg kg^−1^). After that, mice of the two groups were intravenously injected with the Cy5 labeled HPVP (5 mg kg^−1^ of HPVP). The tumor accumulation of HPVP was recorded on an IVIS Spectrum (PerkinElmer) at different time intervals (0.5, 1, 2, 4, 8, 12, and 24 h after the injection). Twenty-four hours after the injection, the tumor-draining lymph nodes were collected and their fluorescence was also recorded by IVIS Spectrum. Besides, the tumor accumulation of HPV16 L1 protein and HPVP nanoparticles of the first injection and second injection were also detected.

### In vivo PDL1 knockdown effect analysis

Subcutaneous 4T1 tumor-bearing mice were respectively administrated with PBS, siRNA1@HPVP, siRNA2@HPVP and siRNA3@HPVP by intravenous injection. Twenty-four hours later, tumor tissues of different groups were obtained and analyzed by immunofluorescence staining or FACS to evaluate the PDL1 expression.

### In vivo TNF-α detection

The as-prepared liposome was mixed with pTNF-α-luc plasmid (with a mass ratio of 1:1.5) and shaken for 5 min for in vivo gene transfection. 4T1 cells (1 × 10^6^ cell per mouse) were subcutaneously injected in the right flank of the mice to build the subcutaneous breast tumor model. When the subcutaneous 4T1 tumor reached about 100 mm^3^, 25 μg of pTNF-α-luc plasmid was intratumoral injected into the mice for gene transfection. Three days after the transfection, the mice were respectively treated with PBS, siRNA, HPVP, and siRNA2@HPVP (equivalent to 5 mg kg^−1^ bodyweight of HPVP and 2.5 mg kg^−1^ bodyweight of siRNA) via *i.v.* injection. At different time points after the injection, the bioluminescence intensity within the tumor was recorded by the IVIS spectrum.

### Transcriptomics study

When the subcutaneous tumor reached about 100 mm^3^, the mice were respectively treated with PBS, αPDL1 (1 mg kg^−1^) and siRNA2@HPVP (7.5 mg kg^−1^) via intratumoral injection. After 3 days, the tumor tissues of all groups were collected, and the high-throughput sequencing was performed in Majorbio BioTech Co., Ltd. The data were analyzed online with I-Sanger Cloud Platform. Vital genes related with the native immune response were analyzed by PCR. Primer sequences are listed in Supplementary table 1.

### Transfection and reporter assays

HEK-293 cells were transiently transfected with TLR homodimers and the ELAM-Luc reporter plasmid by Lipofectamine 2000. Twenty-four hours after the transfection, the cells were stimulated with the fresh medium, siRNA and siRNA2@HPVP for 8 h, respectively. Then, the cells were lysed and the activity of luciferase was examined using the Ultra Sensitive Tube Luminometer (Berthold Lumat LB 9507).

### In vitro TLR7-mediated immunity activation

In this experiment, BMDCs were seeded in a six-well plate (1 × 10^5^ cells per well). After the stimulation with GM-CSF and IL4, the cells were pretreated with hydroxychloroquine sulfate (3.2 mg L^−1^) or fresh medium for 3 h, respectively. After that, the cells with different pretreatments were co-cultured with siRNA2@HPVP (10 mg mL^−1^ siRNA and 20 mg L^−1^ HPVP) or siRNA (10 mg mL^−1^) for another 12 h, and the mature DCs were detected by FACS.

### In vivo immune cells analysis

For mature DCs detection, the subcutaneous 4T1 tumor-bearing mice were respectively treated with PBS, siRNA (2.5 mg kg^−1^), HPVP (5 mg kg^−1^), HPVP plus aPDL1, and siRNA2@HPVP (7.5 mg kg^−1^) via intravenous injection. The tumor-draining lymph nodes were collected 72 h after the treatments. The single cells suspension was obtained by grind, and then co-incubated with anti-CD16/32 antibody (1 μg per tube) for 10 min to block the Fc receptor. Afterward, anti-CD11c (0.25 μg per tube), anti-CD80 (1 μg per tube) and anti-CD86 (1 μg per tube) antibodies were added for staining. For tumor infiltration immune cells analysis, tumor tissues of different groups were collected and digested with the 2 mL of RPMI 1640 medium containing 2% FBS, 1 mg mL^−1^ collagenase IV, 0.1 mg mL^−1^ hyaluronidase, and 0.1 mg mL^−1^ DNase I at 37 °C for 1 h. The obtained single cells suspension was then filtered through a 70-μm nylon cell strainer, and the cells were collected via centrifugation (400 *g*, 5 min) and washed with PBS for three times. For tumor infiltration T cells detection, after blocked with anti-CD16/32 antibody for 10 min, the cells were stained with anti-CD3 (1 μg per tube), anti-CD4 (0.25 μg per tube), and anti-CD8 (1 μg per tube) antibodies. For tumor infiltration macrophage analysis, the cells were stained with anti-CD11b (0.25 μg per tube) and anti-CD206 (5 μg per tube) antibodies after the block process. For the isolation of splenocytes, the spleen tissues of different groups were ground, and the red blood cells lysis was conducted after the cells washed with PBS. Adherent cells were removed by overnight culture. Subsequently, the cells were blocked with anti-CD16/32 antibody for 10 min and then stained with anti-CD8 (1 μg per tube), anti-CD62L (0.25 μg per tube) and anti-CD44 (0.25 μg per tube) antibodies. The staining process was carried out in 1.5 mL EP tube. The cells were dispersed with 100 μL of cell staining buffer (1 × 10^6^ cells each sample), and the incubation was performed at 4 °C for 25 min on the Thermo Shaker Incubator (MIULAB, MTC-100). Before being detected by FACS, the cells were washed with PBS for two times.

### Antitumor effects against the mutational 4T1 tumors

The mutational 4T1 cell lines were obtained through the knockout of *Mlh1* gene. For the knockout of *Mlh1* gene, the genome editing vector system (pSpCas9(BB)-2A-Blasticidin V2.0) was used, and sgRNA (TCACCGTGATCAGGGTGCCC) was designed by the CRISPR tool (http://crispr.mit.edu) to avoid the off-target effect. Then the annealed sgRNA oligonucleotides were cloned into pSpCas9(BB)-2A-Blasticidin V2.0 plasmid. Subsequently, 4T1 cells were transfected with pSpCas9(BB)-2A-Blasticidin V2.0 vector plasmid through the Lipofectamine 3000 for 48 h. After that, cells were incubated with puromycin for 4 days and then single-cell-diluted in 96-well plates. At last, the cell clones lacking of *Mlh1* gene were collected. Mutational statistics of the obtained cell lines were carried out through the SNP, Indel and AS analyses.

Next, we constructed the subcutaneous breast tumor models with the five mutational 4T1 cell lines. After 5 days, the tumor-bearing mice were divided into three groups (*n* = 5), and respectively treated with PBS, aPDL1 plus nabpaclitaxel (1 mg kg^−1^ aPDL1 and 10 mg kg^−1^ nabpaclitaxel) and siRNA2@HPVP plus nabpaclitaxel (7.5 mg kg^−1^ siRNA2@HPVP and 10 mg kg^−1^ nabpaclitaxel). During the therapy, the tumor size and bodyweight were recorded every other day. Two weeks after the treatment, the mice were sacrificed and the tumor tissues were obtained for further analysis. The tumor infiltration T cells were detected with FACS, and the intratumoral immune indexes (CD3, CD4, CD8, Foxp3, IL2, T-bet, and IFN-γ) were evaluated by the on-chip immunofluorescence analysis. Moreover, the mice survival of the four tumor-bearing mouse models with different treatments were also recorded.

### Reporting summary

Further information on experimental design is available in the Nature Research Reporting Summary linked to this paper.

## Supplementary information

Supplementary Information

Description of Additional Supplementary Information

Supplementary Data 1

Reporting Summary

## Data Availability

The source data underlying Figs. 1b, d, g, 2a, c, f, 3d–i, 4a, c, d, f–h, 5d–f, j, 6b–h and Supplementary Figs. 2, 3, 4, 7, and 9 are provided as a Source Data file. Data of the single nucleotide polymorphism annotation of Mlh1-knockout 4T1 cells are included in Supplementary Data 1. The raw data of Fig. 1a and Supplementary Figs 1 are available at http://ualcan.path.uab.edu/index.html and https://www.cancer.gov/about-nci/organization/ccg/research/structural-genomics/tcga. The raw data of Fig. 1b are available at https://www.proteinatlas.org/. All the relevant data are available from the authors upon reasonable request. The transcriptomic data are available at NCBI under Project PRJNA615691 [https://www.ncbi.nlm.nih.gov/sra?LinkName=bioproject_sra_all&from_uid=615691]. A reporting summary for this article is available as a Supplementary Information file.

## Source data

Source Data
