# Supplementary material for: A vaccine-based nanosystem for initiating innate immunity and improving tumor immunotherapy
Source: Nat Commun. 2020 Apr 24;11:1985. doi: 10.1038/s41467-020-15927-0 (PMC7181622; doi:10.1038/s41467-020-15927-0)
Supplement: Supplementary file 1 — Supplementary Information [file 41467_2020_15927_MOESM1_ESM.pdf]

Supplementary Information

A vaccine-based nanosystem for initiating innate immunity and improving tumor immunotherapy

Di-Wei Zheng, Fan Gao, Qian Cheng, Peng Bao, Xue Dong, Jin-Xuan Fan, Wen Song, Xuan Zeng, Si-Xue Cheng and Xian-Zheng Zhang

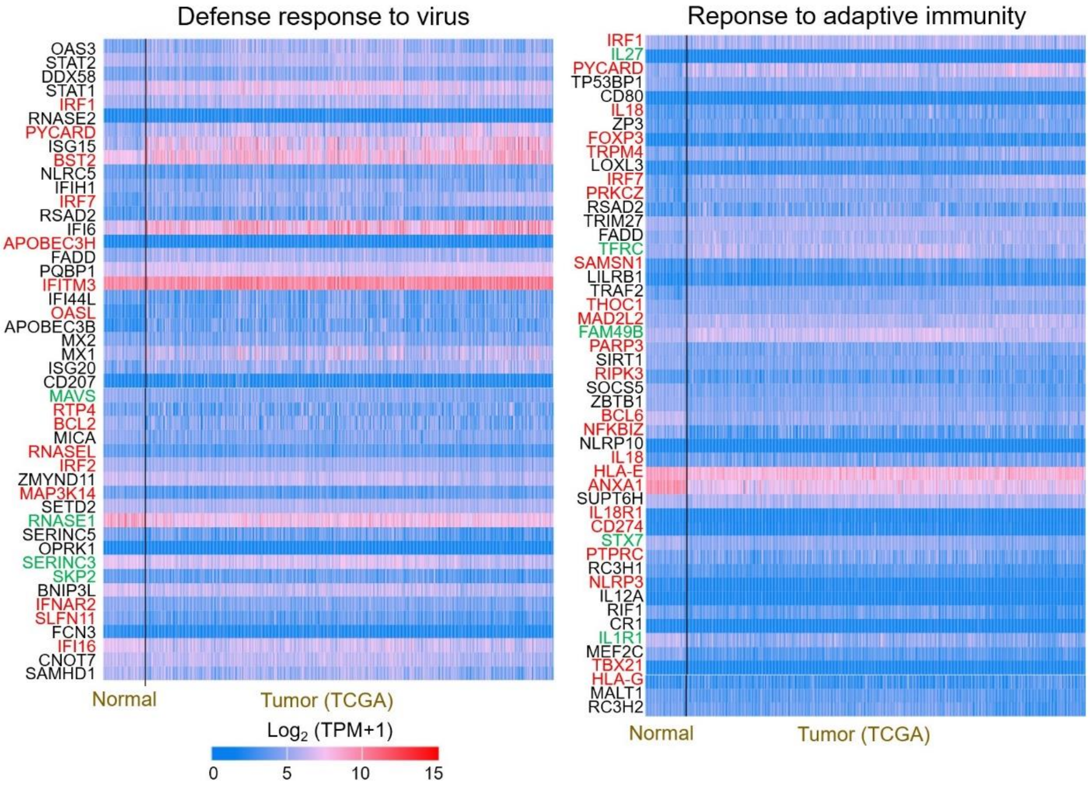

Supplementary Fig. 1 | Clinical data analysis. Heatmap for showing the gene expression data of the virus immunity (left) and adaptive immunity (right) in breast tumor patients from TCGA. The genes labeled red are positively correlated with the prognosis of the patients. The genes labeled green are negatively correlated with the prognosis of the patients. The genes labeled black are not correlated with the prognosis of the patients. Samples from 114 normal breast tissues and 1097 breast tumor tissues were used for in silico analysis.

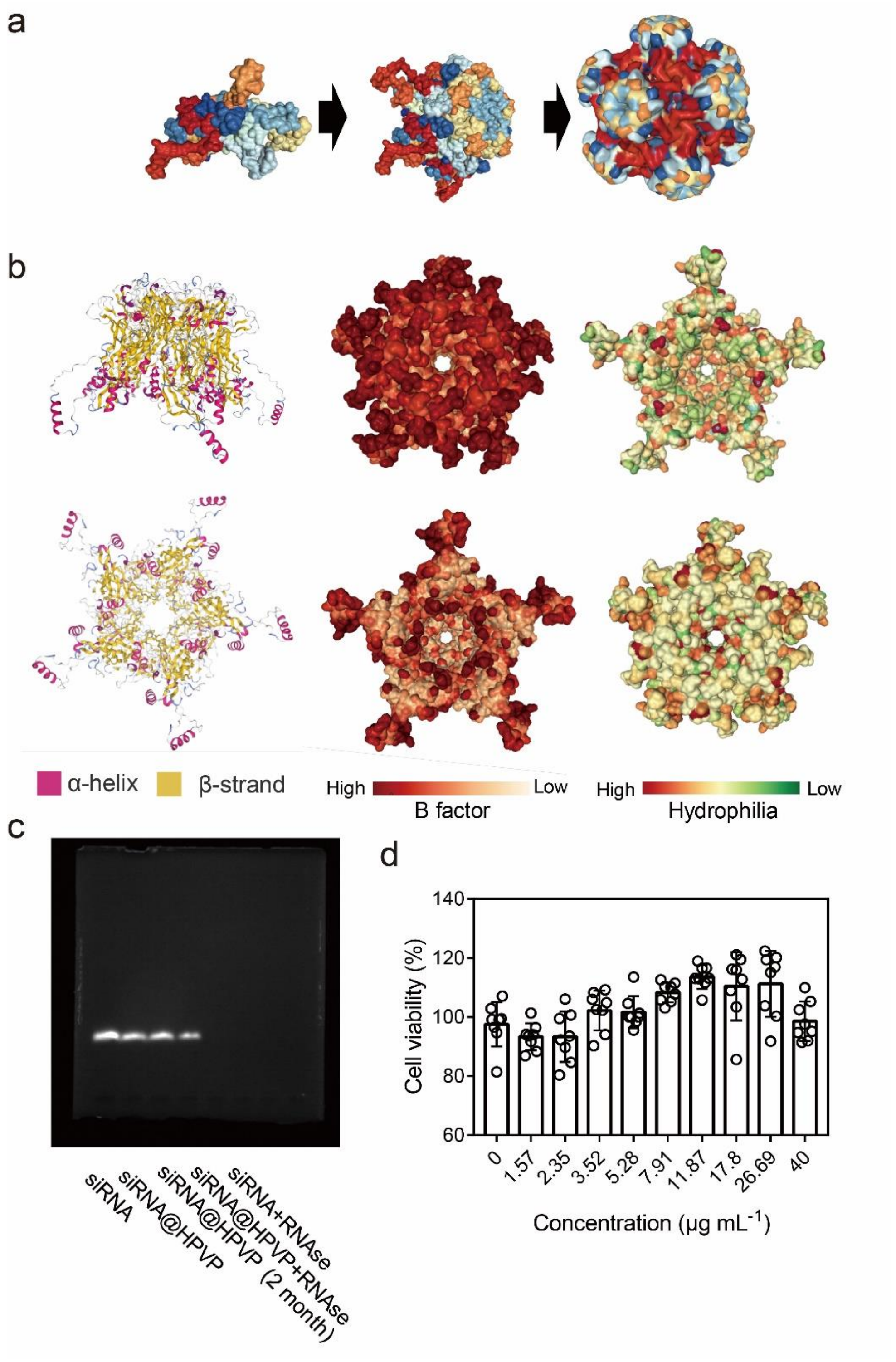

Supplementary Fig. 2 | siRNA@HPVP nanosystem characterization and HPVP structure schematic diagram. (a) Schematic diagram of the monomer, pentamer and assembly morphology of HPVP. (b) Schematic diagram for the secondary structure, B factor and hydrophobic/hydrophilic organization of HPV16 L1 protein assembly. The

protein cavity exhibits high hydrophobicity and positive charge, which may be the driving force for the assembly of this protein with DNA. The protein structure was assigned from Protein Data Bank (http://www.rcsb.org/). (c) The protective capacity of HPVP for siRNA from enzymatic degradation or long-term storage. 5 mU of RNase I was for initiating the enzymatic RNA degradation (15 min). The stock solution (0.8 mg mL^{-1}) of siRNA@HPVP was stored at -20 °C for 2 month. (d) The cytotoxicity of HPVP towards 4T1 cells. Eight biological replicates are shown. Data are presented as mean values +/- SD.

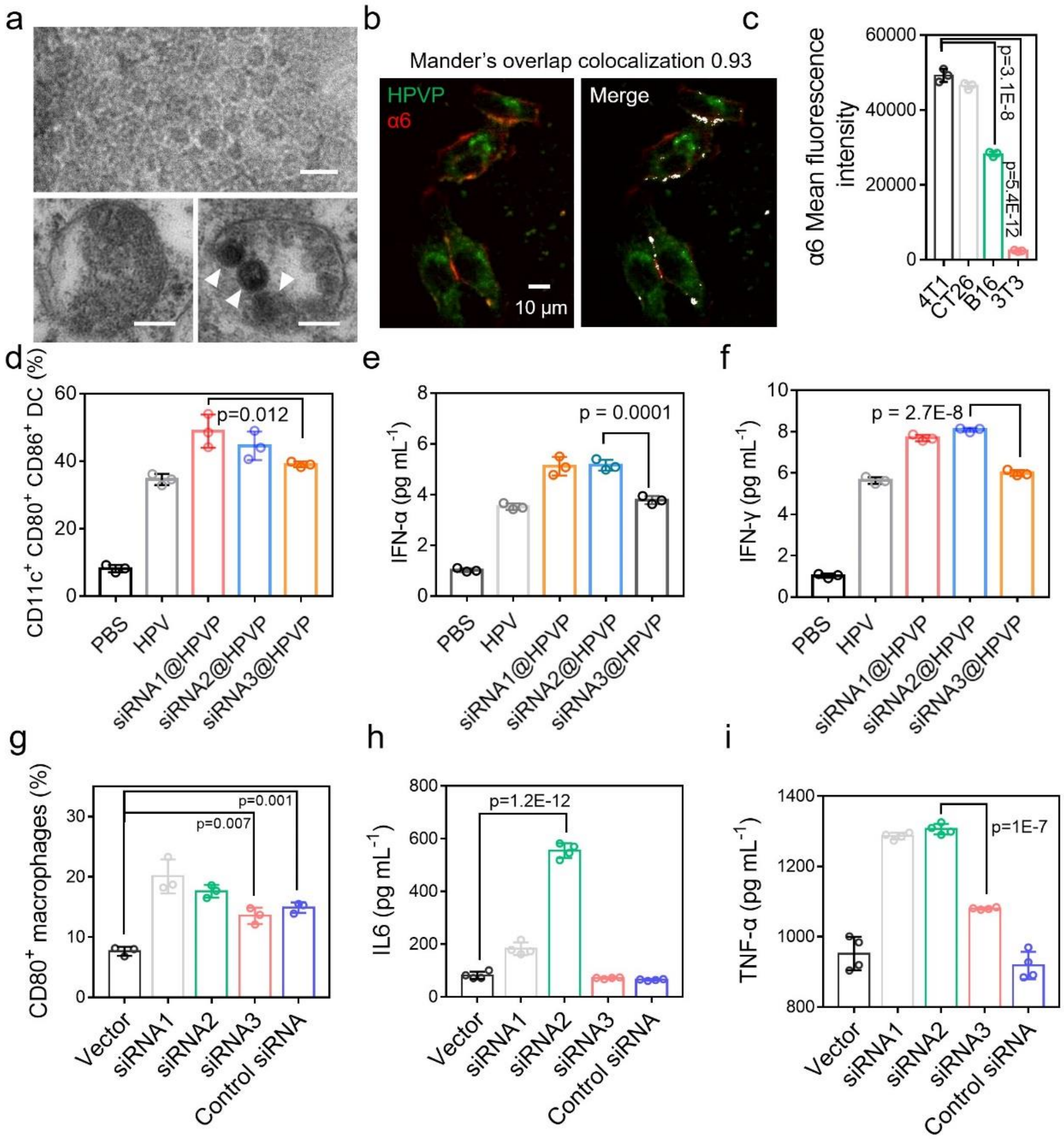

Supplementary Fig. 3 | *In vitro* study of siRNA@HPVP. (a) TEM images of siRNA@HPVP (Scale bar: 75 μm). Morphology of siRNA@HPVP in aqueous solution (up) and intracellular environment (down). Three images per group were taken. (b) Fluorescence co-localization experiment of HPVP and α6 integrin in 4T1 cells. Mander's R^2 and Pearson's coefficients were calculated with the "Colocalization Finder" plugin for ImageJ. A representative image of two biological replicates is shown. (c) Quantitative FACS study of the α6 integrin expression on 4 cell lines (4T1, B16, CT26 and 3T3 cell lines). Both 4T1 and CT26 cells exhibit high α6 integrin expression level

(n = 3 biologically independent samples). (d) FACS examination of the mature DCs phenotype ($CD11c^+CD80^+CD86^+$) proportion after stimulated with PBS, HPVP (20 mg L^{-1}), siRNA1@HPVP (30 mg L^{-1}), siRNA2@HPVP (30 mg L^{-1}), and siRNA3@HPVP (30 mg L^{-1}) for 24 h. Three biological replicates are shown. (e) and (f) The cytokines (IFN-α and IFN-γ) released by mature DC cells in supernatant were also quantified *via* ELISA assay. Three biological replicates are shown. (g) FACS examination of the activation of macrophages after stimulated with different siRNA sequences (5 mg L^{-1}) for 24 h. Three biological replicates are shown. (h) and (i) The cytokines (TNF-α and IL6) released during macrophages activation were also quantified *via* ELISA assay (n = 3 biologically independent samples). Statistical significance was calculated via one-way ANOVA with a Tukey post-hoc test (c, d, e, f, g, h, i). Data are presented as mean values +/- SD.

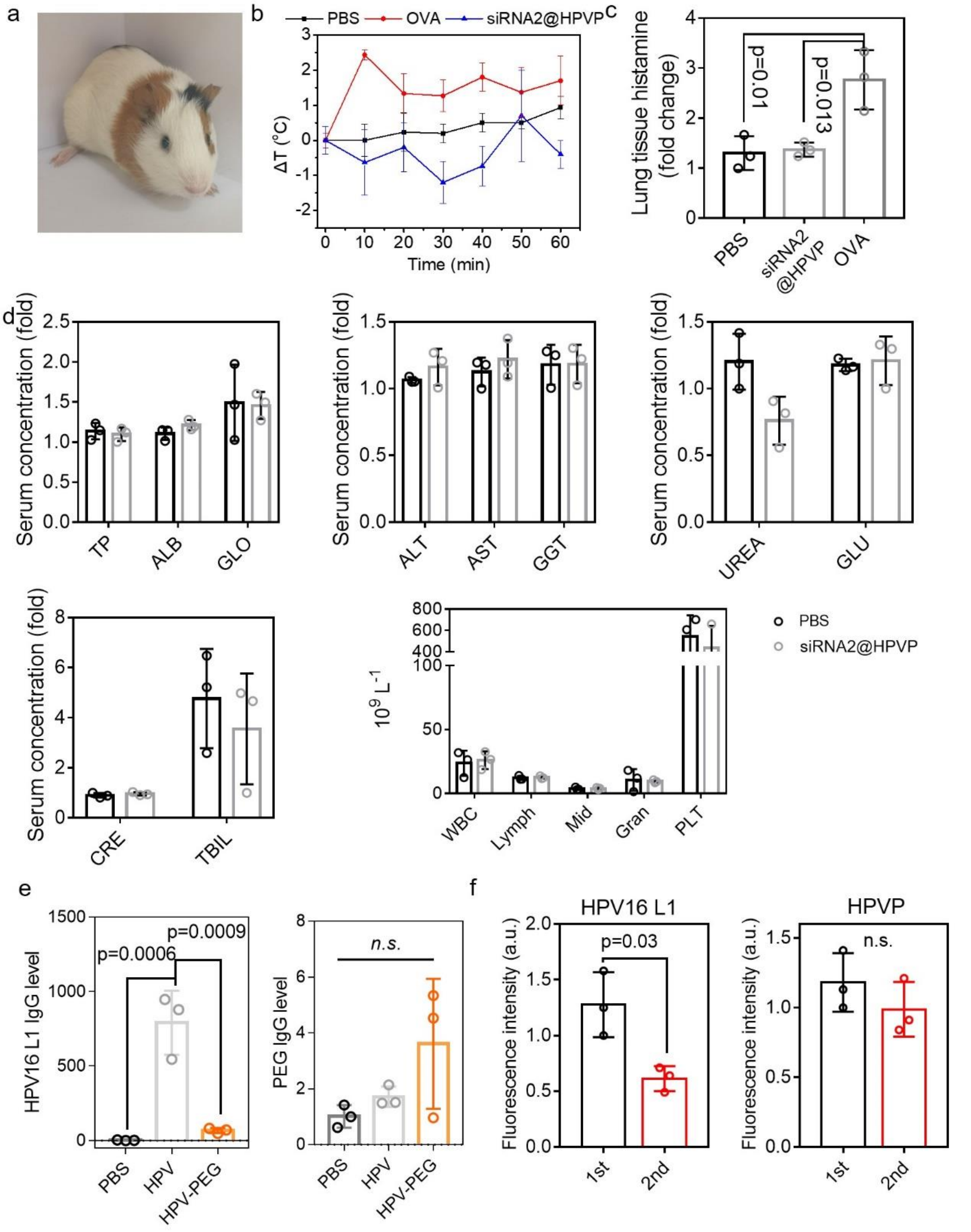

Supplementary Fig. 4 | Biosafety and allergy test. (a) Digital photo of laboratory guinea pig. (b) Body temperature changes within 60 min after subcutaneous injection with siRNA2@HPVP (500 µg per mouse). The same dose of PBS and ovalbumin (OVA, 500 µg per mouse) group were also performed as the negative control and positive

control. Three biological replicates are shown. (c) Histamine content in lung tissue after treated with various agents as indicated. Three biological replicates are shown. Statistical significance was calculated via one-way ANOVA with a Tukey post-hoc test. (d) Blood biochemistry and blood routine assays were conducted 3 days post injection. Blood biochemistry parameters include the contents of total protein (TP), albumin (ALB), globulin (GLO), alanine aminotransferase (ALT), aspartate aminotransferase (AST), γ-glutamyl transpeptidase (GGT), urea nitrogen (UREA), glucose (GLU), creatinine (CRE) and bilirubin (TBIL). Blood routine parameters include white blood cell (WBC) counts, lymphocyte (Lymph) counts, intermediate cell (Mid) counts, granulocyte (Gran) counts and platelet (PLT) counts. The negligible fluctuations of the blood parameters demonstrated the good biosafety of siRNA2@HPVP. Three biological replicates are shown. (e) The amount of antibody produced in the serum after two immunizations. (f) The effect of repeated administration on the tumor-targeting effect of HPVP and HPV16 L1. The fluorescence intensity of Cy5 in the tumor was detected after homogenization. The time interval between two injections was 5 days. Three biological replicates are shown. Statistical significance was calculated with two-tailed Student's t-test (e, f). Data are presented as mean values +/- SD.

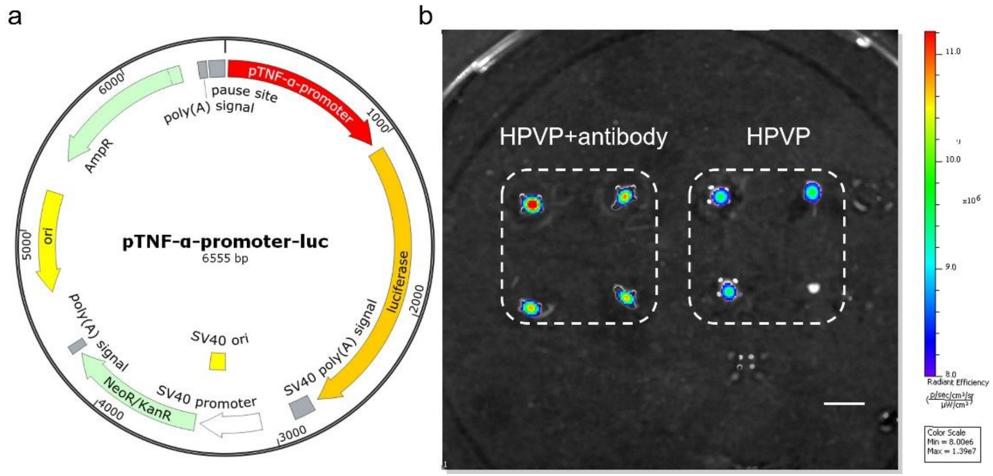

Supplementary Fig. 5 | *In vivo* imaging study. (a) The plasmid structure of pTNF-α-promoter-luc. (b) *Ex vivo* fluorescence imaging of tumor draining lymph node 24 h after the injection (Scale bar: 5 mm). HPVP was labeled with Cy5, and then intravenously injected (5 mg kg^{-1}) into the mice with the presence or absence of α6 integrin antibody. The introduced antibody was used to block the interaction between α6 integrin and HPVP, which might reduce the retention of HPVP in tumor site, and more HPVP entered lymph node. A representative image of three biological replicates is shown.

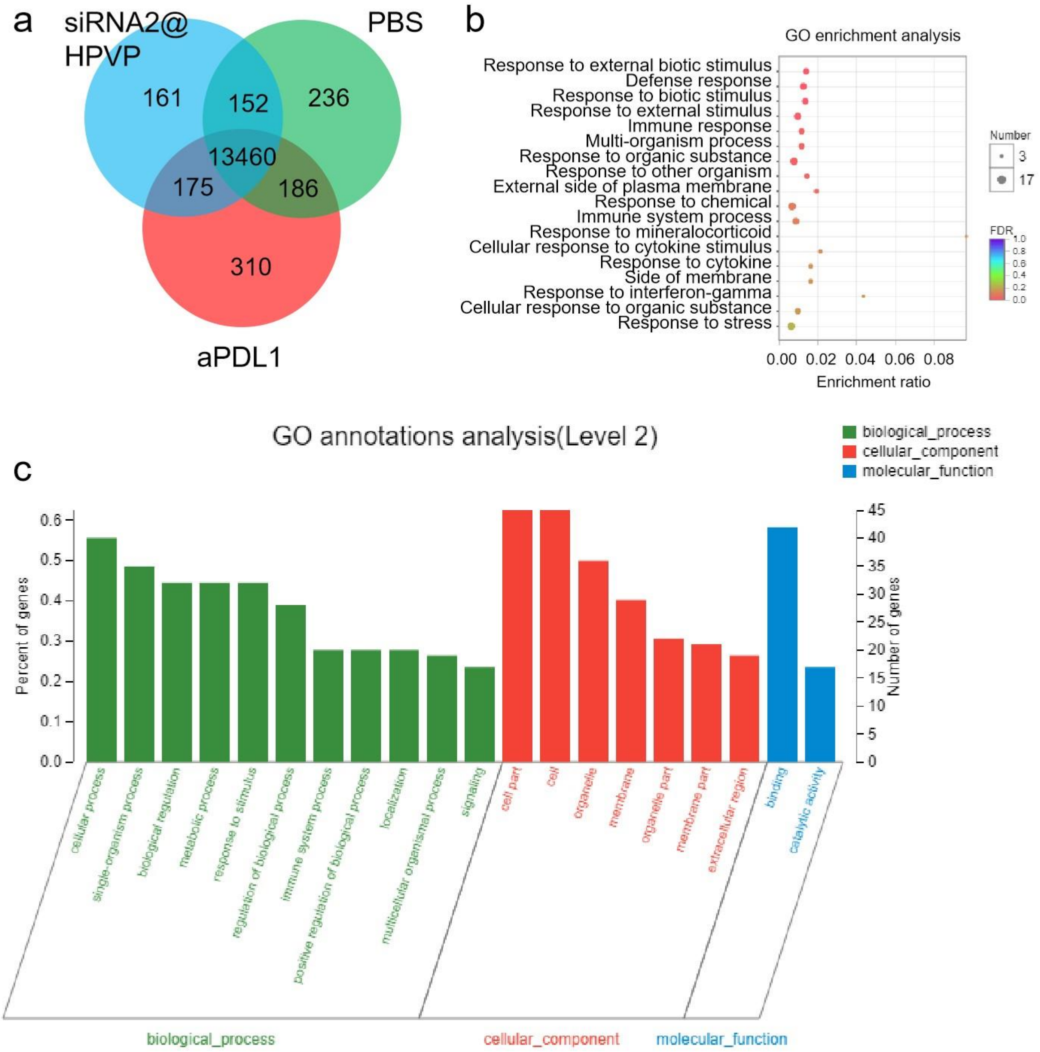

Supplementary Fig. 6 | Transcriptomics analysis of the tumors from siRNA2@HPVP treated mice. (a) Venn diagram for the differentially expressed genes detected at siRNA2@HPVP, aPDL1 or PBS treated mice tumors. (b) GO enrichment analysis of differentially expressed genes between siRNA2@HPVP and PBS treated mice tumors. (c) GO enrichment analysis of identified genes in siRNA2@HPVP, aPDL1 and PBS treated mice tumors.

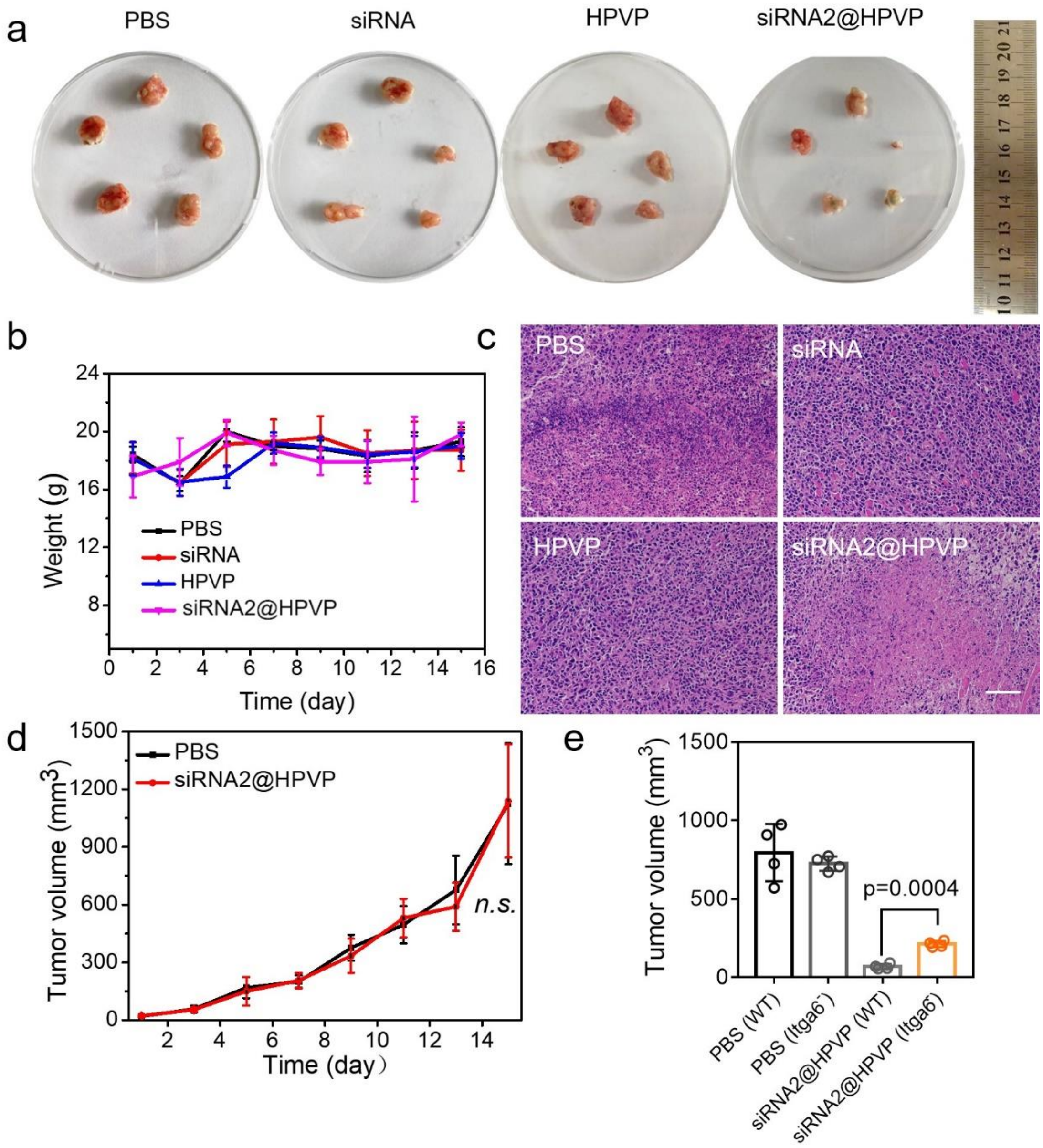

Supplementary Fig. 7 | Anti-tumor effect evaluation of siRNA@HPVP treatment on 4T1 subcutaneous tumor model. (a) At day 15 of different treatments, mice were euthanized and the tumor tissues were excised and photographed for visualization of the tumor size. (b) Body weight changes of the treated mice during the therapy. Five biological replicates are shown. (c) H&E images of tumor tissues (Scale bar: 100 μm). A representative image of three biological replicates is shown. (d) The therapeutic effect of siRNA@HPVP on 4T1 tumor-bearing NOD/SCID mice. 7.5 mg kg^{-1} of

siRNA2@HPVP was given once a week. Six biological replicates are shown. (**e**) The therapeutic effect of siRNA@HPVP was α6-integrin-dependent. 7.5 mg kg^{-1} of siRNA2@HPVP was given once a week. The tumor volume of PBS treated 4T1(WT) tumor, PBS treated $4T1^{Itga6-}$ tumor, siRNA2@HPVP treated $4T1^{Itga6-}$ tumor, and siRNA2@HPVP treated 4T1(WT) tumor at day 15 is shown. Four biological replicates are shown. Statistical significance was calculated with two-tailed Student's t-test (d) and one-way ANOVA with Tukey post-hoc test (e). Data are presented as mean values +/- SD.

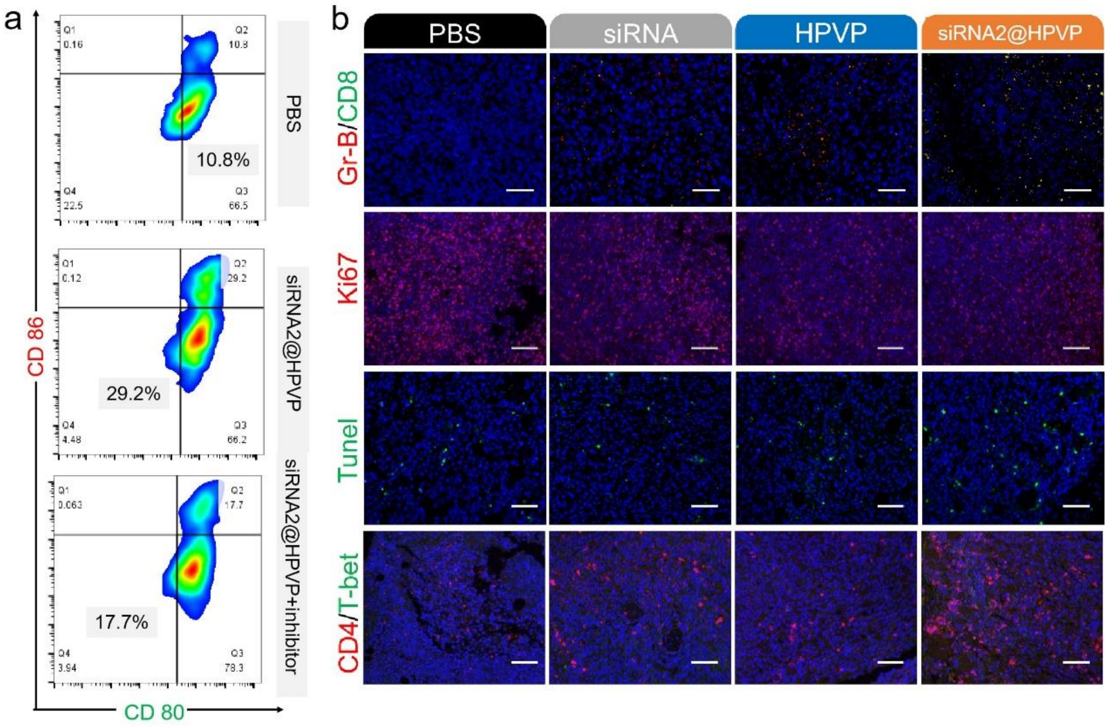

Supplementary Fig. 8 | Immune indexes and TLR7-blockade immune analysis. (a) Hydroxychloroquine sulfate (3.2 mg L^{-1}), the inhibitor of TLR, prevented the maturation of dendritic cells stimulated by siRNA2@HPVP. A representative image of three biological replicates is shown. (b) The mice were subcutaneously injected with PBS, siRNA (2.5 mk kg^{-1}), HPVP (5 mg kg^{-1}) and siRNA2@HPVP (7.5 mg kg^{-1}). Tumor tissues were collected 15 days after different treatments. The mice treated by siRNA2@HPVP could obviously recruit the granzyme B (Gr-B)-secreting tumor infiltration cytotoxic T cells ($CD8^+Gr\text{-}B^+$) and T_H1 cells ($CD4^+T\text{-}bet^+$), resulting in superior anti-tumor effect according to the higher apoptotic area and reduced proliferative activity in siRNA2@HPVP group than other groups (Scale bar: 100 μm). A representative image of three biological replicates is shown.

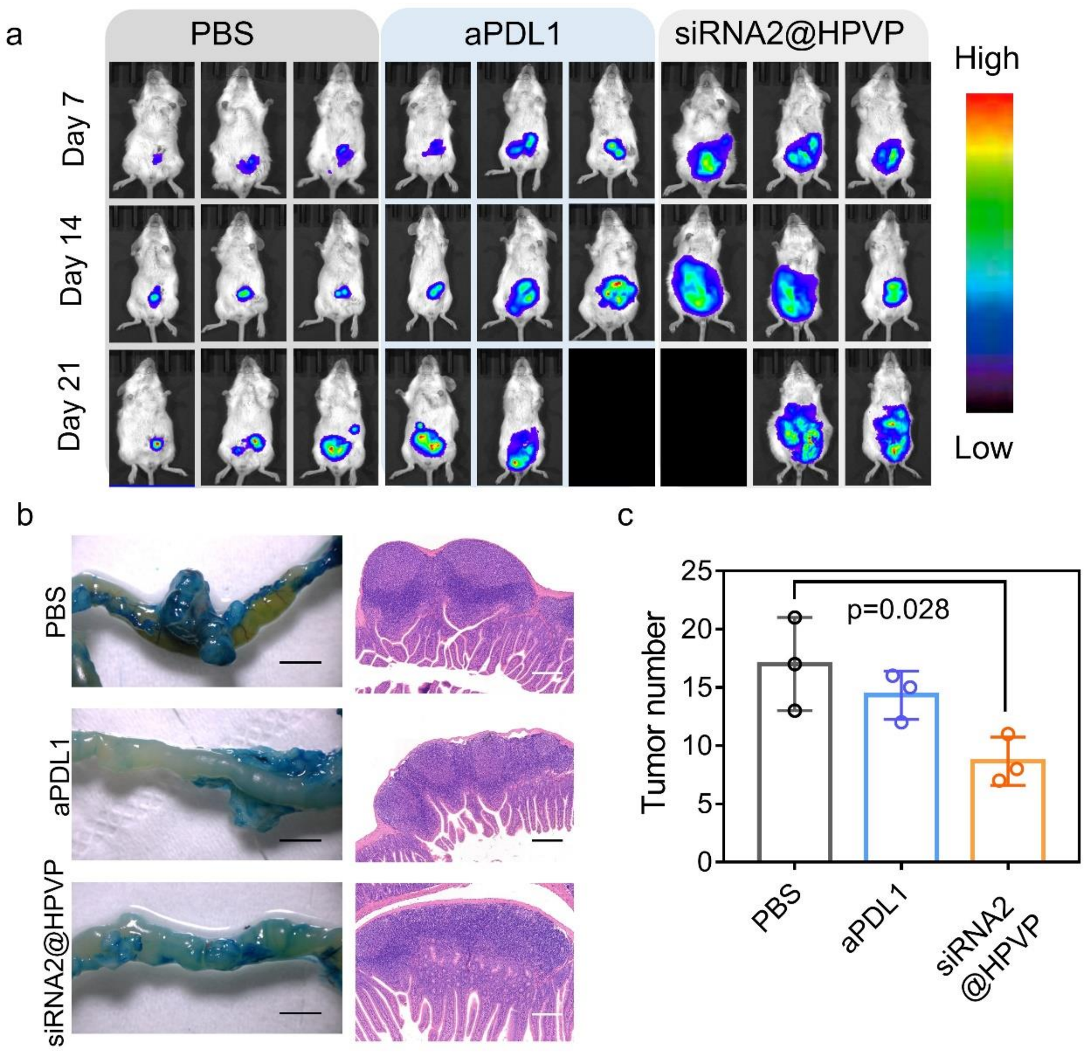

Supplementary Fig. 9 | Anti-tumor effect evaluation of siRNA2@HPVP treatment on orthotopic CT26 tumor-bearing mice. (a) *In vivo* bioluminescence images of orthotopic colon tumor-bearing mice after different treatments. Three representative images of six biological replicates are shown. (b) Pathological observation of orthotopic tumors. At last day of the therapy, intestinal tissues of different groups were collected and stained with H&E (A representative image of three biological replicates is shown) to observe the tumor invasion (left) (Scale bar: left: 3 mm; right: 200 μm). (c) Quantitative analysis of the number of tumors *in situ*. The invasive tumor nodules were quantified through the H&E images (right, three biological replicates are shown).

Statistical significance was calculated *via* one-way ANOVA with a Tukey post-hoc test.

Data are presented as mean values +/- SD.

	1-5	6-10	11-15
A	L2-PBS	L2-aPDL1	L2-s@H
B	L3-PBS	L3-aPDL1	L3-s@H
C	L4-PBS	L4-aPDL1	L4-s@H
D	L5-PBS	L5-aPDL1	L5-s@H
E	L2-PBS	L2-aPDL1	L2-s@H
F	L3-PBS	L3-aPDL1	L3-s@H
G	L4-PBS	L4-aPDL1	L4-s@H
H	L5-PBS	L5-aPDL1	L5-s@H

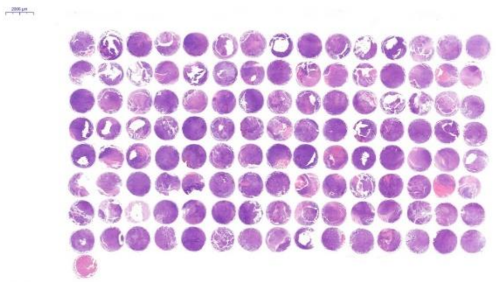

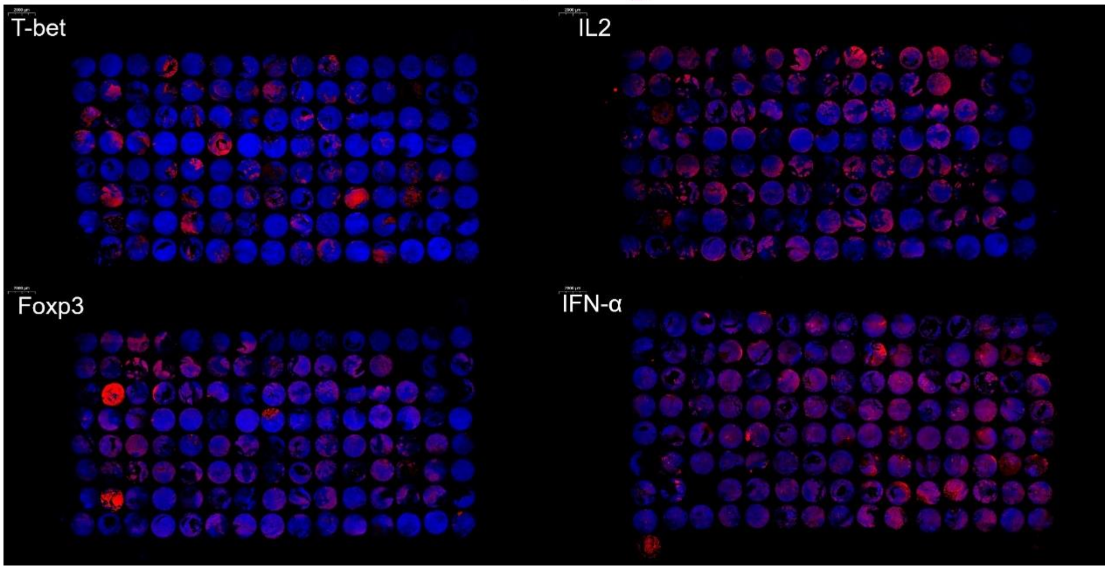

Supplementary Fig. 10 | The therapeutic effect of siRNA@HPVP in 4T1^{mut} tumor-bearing mice. On-chip immunohistochemical analysis of 4T1^{mut} tumor**.** At the day 15 of the treatment, the mice were sacrificed and 4T1^{mut-2}, 4T1^{mut-3}, 4T1^{mut-4} and 4T1^{mut-5} tumor tissues were collected for H&E staining and immune indexes detection. H&E staining was explored to evaluate the anti-tumor effect. The immunofluorescence assays of T-bet, IL2, Foxp3 and IFN-α were carried out to characterize the immune stimulate effects of different treatments. The nomenclature in the table is according to the following rules. L2-L5 respectively represent the tumor tissues obtained from 4T1^{mut-2}, 4T1^{mut-2}, 4T1^{mut-3}, 4T1^{mut-4} and 4T1^{mut-5} tumor-bearing mice. PBS, aPDL1 and s@H (siRNA2@HPVP) respectively represent different treatments.

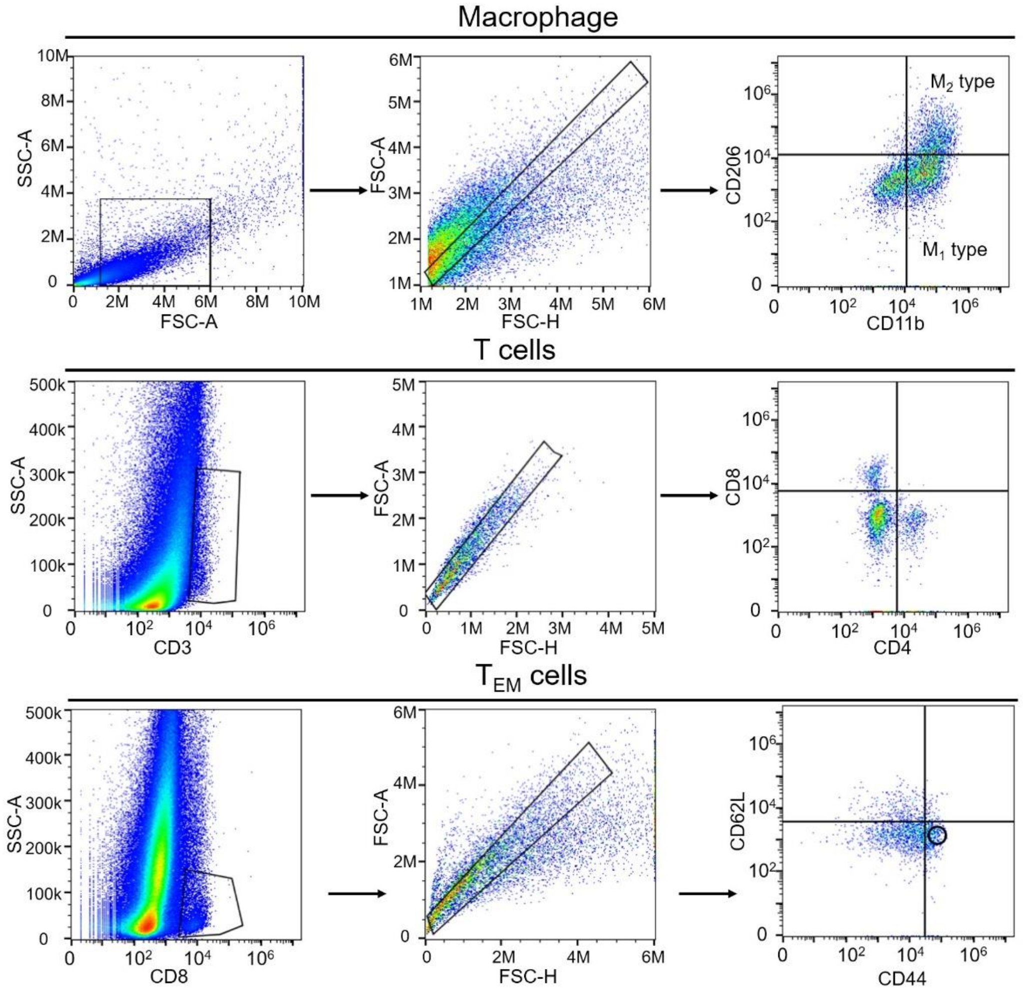

Supplementary Fig. 11 | Gating strategies of immune cells in tissues. All cells statistics were conducted with the single cell format. CD11b$^+$CD206$^-$ cells and CD11b$^+$CD206$^+$ cells are respectively defined as M1 macrophages and M2 macrophages. CD3$^+$CD8$^+$ cells are defined as cytotoxic T cells. CD3$^+$CD4$^+$ cells are defined as T helper cells. Both macrophages and T cells were detected in tumor tissues, while the T_{EM} cells (CD8$^+$CD44$^+$CD62$^-$) were detected in spleen tissues.

Supplementary Table 1 | Primer sequences.

Gene	Sequence (5'-3')
Tlr7	GTACCAAGAGGCTGCAGATTAGAC (F)
	AGCCTCAAGGCTCAGAAGATG (R)
Cxcl9	TCCTTTTGGGCATCATCTTCC (F)
	TTTGTAGTGGATCGTGCCTCG (R)
Ifnb1	CAGCTCCAAGAAAGGACGAAC (F)
	GGCAGTGTAACTCTTCTGCAT (R)
Ifngr1	CTGGCAGGATGATTCTGCTGG (F)
	GCATACGACAGGGTTCAAGTTAT (R)
Ifng	ATGAACGCTACACACTGCATC (F)
	CCATCCTTTTGCCAGTTCCTC (R)
Gapdh	ATGGGTGTGAACCACGAGA (F)
	CAGGGATGATGTTCTGGGCA (R)
Cd274	CTGCCAAAGGACCAGCTTTT (F)
	GGCTGGATCCACGGAAATTC (R)
