# Supplementary material for: A vaccine-based nanosystem for initiating innate immunity and improving tumor immunotherapy
Source: Nat Commun. 2020 Apr 24;11:1985. doi: 10.1038/s41467-020-15927-0 (PMC7181622; doi:10.1038/s41467-020-15927-0)
Supplement: Supplementary file 2 — Description of Additional Supplementary Information [file 41467_2020_15927_MOESM2_ESM.pdf]

Description of Additional Supplementary Files

File Name: Supplementary Data 1
Description: Data of the single nucleotide polymorphism annotation of Mlh1-knockout 4T1 cells.
