# Supplementary material for: A vaccine-based nanosystem for initiating innate immunity and improving tumor immunotherapy
Source: Nat Commun. 2020 Apr 24;11:1985. doi: 10.1038/s41467-020-15927-0 (PMC7181622; doi:10.1038/s41467-020-15927-0)
Supplement: Supplementary file 4 — Reporting Summary [file 41467_2020_15927_MOESM4_ESM.pdf]

naturerresearch

Corresponding author(s): Xian-Zheng Zhang

Last updated by author(s): Mar 11, 2020

Reporting Summary

Nature Research wishes to improve the reproducibility of the work that we publish. This form provides structure for consistency and transparency in reporting. For further information on Nature Research policies, see Authors & Referees and the Editorial Policy Checklist .

Statistics

For all statistical analyses, confirm that the following items are present in the figure legend, table legend, main text, or Methods section.

n/a	Confirmed	
☐	☒	The exact sample size (*n*) for each experimental group/condition, given as a discrete number and unit of measurement
☐	☒	A statement on whether measurements were taken from distinct samples or whether the same sample was measured repeatedly
☐	☒	The statistical test(s) used AND whether they are one- or two-sided<br>*Only common tests should be described solely by name; describe more complex techniques in the Methods section.*
☐	☒	A description of all covariates tested
☐	☒	A description of any assumptions or corrections, such as tests of normality and adjustment for multiple comparisons
☐	☒	A full description of the statistical parameters including central tendency (e.g. means) or other basic estimates (e.g. regression coefficient) AND variation (e.g. standard deviation) or associated estimates of uncertainty (e.g. confidence intervals)
☒	☐	For null hypothesis testing, the test statistic (e.g. *F*, *t*, *r*) with confidence intervals, effect sizes, degrees of freedom and *P* value noted<br>*Give P values as exact values whenever suitable.*
☒	☐	For Bayesian analysis, information on the choice of priors and Markov chain Monte Carlo settings
☒	☐	For hierarchical and complex designs, identification of the appropriate level for tests and full reporting of outcomes
☒	☐	Estimates of effect sizes (e.g. Cohen's *d*, Pearson's *r*), indicating how they were calculated

Our web collection on statistics for biologists contains articles on many of the points above.

Software and code

Policy information about availability of computer code

Data collection	No software was used.
Data analysis	All statistical analyses were performed on Origin (version 8.6), SPSS (version 22) or Excel 2016. Living image software (Version 4.5) was used to analyse bioluminescent and fluorescent images. Image J (Version 1.48h3) was used for fluorescence-image analysis. Transcriptomics data were analyzed online with I-Sanger Cloud Platform (https://cloud.majorbio.com). Gensys (Version1.6.9.0) was used to image the agarose gel. FlowJo (Version 4.5) was used for flow cytometry analysis.

For manuscripts utilizing custom algorithms or software that are central to the research but not yet described in published literature, software must be made available to editors/reviewers. We strongly encourage code deposition in a community repository (e.g. GitHub). See the Nature Research guidelines for submitting code & software for further information.

Data

Policy information about availability of data

All manuscripts must include a data availability statement. This statement should provide the following information, where applicable:

- Accession codes, unique identifiers, or web links for publicly available datasets
- A list of figures that have associated raw data
- A description of any restrictions on data availability

The source data underlying Figs. 1b, d, g, 2a, c, f, 3d-i 4a, c-d, f-h 5d-f, j, 6b-h and Supplementary Figs 2, 3, 4, 7, and 9 are provided as a Source Data file. The raw data of Fig. 1a and Supplementary Figs 1 are available at http://ualcan.path.uab.edu/index.html, and https://www.cancer.gov/about-nci/organization/ccg/research/structural-genomics/tcga. The raw data of Fig. 1b are available at https://www.proteinatlas.org/. All the relevant data are available from the authors upon reasonable request. A reporting summary for this article is available as a Supplementary Information file.

Field-specific reporting

Please select the one below that is the best fit for your research. If you are not sure, read the appropriate sections before making your selection.

☒ Life sciences ☐ Behavioural & social sciences ☐ Ecological, evolutionary & environmental sciences

For a reference copy of the document with all sections, see nature.com/documents/nr-reporting-summary-flat.pdf

Life sciences study design

All studies must disclose on these points even when the disclosure is negative.

Sample size	No sample size calculation was performed. Sample sizes were determined according to previous experimental experience. The sample sizes of in vitro experiments refers to previously published literature (Nature Nanotechnology, 2019, 14, 89-97). The sample sizes of in vivo experiments refers to previously published literature (Nature Biomedical Engineering, 2018, 2, 611-621).
Data exclusions	No data were excluded.
Replication	All experimental data are given including replicates. Details of experimental replicates are given in the figure legends. All reported attempts at replication were successful.
Randomization	All samples/organisms were randomly allocated into experimental groups.
Blinding	Blinding and randomization were applied to all experiments.

Reporting for specific materials, systems and methods

We require information from authors about some types of materials, experimental systems and methods used in many studies. Here, indicate whether each material, system or method listed is relevant to your study. If you are not sure if a list item applies to your research, read the appropriate section before selecting a response.

Materials & experimental systems

n/a	Involved in the study	
☐	☒	Antibodies
☐	☒	Eukaryotic cell lines
☒	☐	Palaeontology
☐	☒	Animals and other organisms
☒	☐	Human research participants
☒	☐	Clinical data

Methods

n/a	Involved in the study	
☒	☐	ChIP-seq
☐	☒	Flow cytometry
☒	☐	MRI-based neuroimaging

Antibodies

Antibodies used

Anti-mouse IFNAR-1 Antibody (Bioxcell, Cat. NO. BE0241, Clone: MAR1-5A3)
Anti-mouse PD-L1 Antibody (Bioxcell, Cat. NO. BE0101, Clone: 10F.9G2)
Anti-mouse CD16/32 Antibody (Biolegend, CAT.NO. 101329, Clone: 93)
FITC Anti-mouse CD3 Antibody (Biolegend, CAT.NO. 100203, Clone: 17A2)
PE Anti-mouse CD4 Antibody (Biolegend, CAT.NO. 100407, Clone: GK1.5)
APC Anti-mouse CD8a Antibody (Biolegend, CAT.NO. 100711, Clone: 53-6.7)
PE Anti-mouse CD11c Antibody (Biolegend, CAT.NO. 117307, Clone: N418)
FITC Anti-mouse CD80 Antibody (Biolegend, CAT.NO. 104705, Clone: 16-10A1)
APC Anti-mouse CD86 Antibody (Biolegend, CAT.NO. 105011, Clone: GL-1)
PE Anti-mouse/human CD11b Antibody (Biolegend, CAT.NO. 101207, Clone: M1/70)
APC Anti-mouse CD206 Antibody (Biolegend, CAT.NO. 141707, Clone: C068C2)
FITC Anti-mouse CD8a Antibody (Biolegend, CAT.NO. 100705, Clone: 53-6.7)
APC Anti-mouse/human CD44 Antibody (Biolegend, CAT.NO. 103011, Clone: IM7)
PE Anti-mouse CD62L Antibody (Biolegend, CAT.NO. 104407, Clone: MEL-14)
IFN-α polyclonal Antibody (Bioss Guarantee, CAT.NO. bs-6304R, Clone: Polyclonal)
Anti-IFN-γ (Abcam, CAT.NO. ab9657)
HRP-labeled goat Anti-mouse IgG (Beyotime, CAT.NO: A0216, Source: Goat)
Anti-T-bet (Santa Cruz Biotechnology, CAT.NO. sc-21749, Source: recombinant murine)
Anti-IL-2 Rabbit pAb (GB11114, CAT.NO. GB11114)
Mouse IL6 Elisa Kit (4A Biotech Co., Ltd. CAT.NO. CME0006)

	Mouse IL1β Elisa Kit (4A Biotech Co., Ltd. CAT.NO. CME0015)<br>Mouse TNF-α Elisa Kit (4A Biotech Co., Ltd. CAT.NO. CME0004)<br>Mouse IFN-γ Elisa Kit (4A Biotech Co., Ltd. CAT.NO. CME0003-10*96)<br>Mouse IFN-α Elisa Kit (Solarbio, CAT.NO. SEKM-0149)<br>Mouse MIP1α Elisa Kit (USCN Business Co., Ltd. CAT.NO. SEA092Mu)<br>Histamine Elisa Kit (USCN Business Co., Ltd. CAT.NO. CEA927Ge)
Validation	All antibodies were validated by the commercial supplier. All validation statements can be found on the respective antibody website:<br>Anti-mouse IFNAR-1 Antibody: https://bxcell.com/product/Anti-m-ifnar-1/<br>Anti-mouse PD-L1 Antibody: https://bxcell.com/product/m-pdl-1/<br>Anti-mouse CD16/32 Antibody: https://www.biolegend.com/en-us/products/ultra-leaf-purified-Anti-mouse-cd16-32-Antibody-8081<br>FITC Anti-mouse CD3 Antibody: https://www.biolegend.com/en-us/products/fitc-Anti-mouse-cd3-Antibody-45<br>PE Anti-mouse CD4 Antibody: https://www.biolegend.com/en-us/products/pe-Anti-mouse-cd4-Antibody-250<br>APC Anti-mouse CD8a Antibody: https://www.biolegend.com/en-us/products/pe-Anti-mouse-cd4-Antibody-250<br>PE Anti-mouse CD11c Antibody: https://www.biolegend.com/en-us/products/pe-Anti-mouse-cd11c-Antibody-1816<br>FITC Anti-mouse CD80 Antibody: https://www.biolegend.com/en-us/products/fitc-Anti-mouse-cd80-Antibody-41<br>APC Anti-mouse CD86 Antibody: https://www.biolegend.com/en-us/products/apc-Anti-mouse-cd86-Antibody-2896<br>PE Anti-mouse/human CD11b Antibody: https://www.biolegend.com/en-us/search-results?PageSize=25&Keywords=CD11b&k1=CD11b&Reactivity=mouse&Format=FITC&Format=PE<br>APC Anti-mouse CD206 Antibody: https://www.biolegend.com/en-us/products/apc-Anti-mouse-cd206-mmr-Antibody-7425<br>FITC Anti-mouse CD8a Antibody: https://www.biolegend.com/en-us/products/fitc-Anti-mouse-cd8a-Antibody-153<br>APC Anti-mouse/human CD44 Antibody: https://www.biolegend.com/en-us/products/apc-Anti-mouse-human-cd44-Antibody-312<br>PE Anti-mouse CD62L Antibody: https://www.biolegend.com/en-us/products/pe-Anti-mouse-cd62l-Antibody-386<br>IFN-α polyclonal Antibody: https://www.biossusa.com/products/bs-6304r<br>Anti-IFN-γ: https://www.abcam.cn/interferon-gamma-Antibody-ab9657.html<br>HRP-labeled goat Anti-mouse IgG: https://www.beyotime.com/product/A0216.htm<br>Anti-T-bet: https://www.scbt.com/zh/p/t-bet-Antibody-4b10?requestFrom=search<br>Anti-IL-2 Rabbit pAb: http://www.servicebio.cn/html/all/cp/kt/yk/1392.html<br>Mouse IL6 Elisa Kit: http://www.4abio.net/OtherProducts/CME0006<br>Mouse IL1β Elisa Kit: http://www.4abio.net/OtherProducts/CME0015<br>Mouse TNF-α Elisa Kit: http://www.4abio.net/OtherProducts/CME0004<br>Mouse IFN-γ Elisa Kit: http://www.4abio.net/OtherProducts/CME0003-10*96<br>Mouse IFN-α Elisa Kit: http://www.solarbio.com/search.php?encode=YTo0OntzOjg6ImNhdGVnb3J5IjtzOjE6IjAiO3M6ODoia2V5d29yZHMiO3M6NToiSWZOLWEiO3M6NjoiZGF0YUJpIjtzOjI6ImsxIjtzOjE4OiJzZWFyY2hfZW5jb2RlX3RpbWUiO2k6MTU4MzkyMjczNDt9<br>Mouse MIP1α Elisa Kit: http://www.uscnk.com/uscn/ELISA-Kit-for-Mouse-Macrophage-Inflammatory-Protein-1-Alpha-MIP1a-CCL3-1894.htm<br>Histamine Elisa Kit: http://www.uscnk.com/uscn/ELISA-Kit-for-Histamine-HIS-2200.htm

Eukaryotic cell lines

Policy information about cell lines

Cell line source(s)	3T3 mouse embryonal fibroblasts, B16 murine melanoma cells, 4T1 murine breast tumor cells, CT26 murine colon tumor cells, Raw 267.4 murine macrophages and HEK293 cells were obtained from China Center for Type Culture Collection (CCTCC).
Authentication	The cell lines were not authenticated.
Mycoplasma contamination	All cell lines were tested for mycoplasma contamination. No mycoplasma contamination was found.
Commonly misidentified lines (See ICLAC register)	No commonly misidentified cell lines were used.

Animals and other organisms

Policy information about studies involving animals; ARRIVE guidelines recommended for reporting animal research

Laboratory animals	Female C57BL/6 mice (6 weeks), female BALB/c mice (6 weeks), and female Dunkin-Hartley guinea pigs (4 weeks) were used. Animals were housed in groups of 4-6 mice per individually ventilated cage in a 12 h light dark cycle (06:30-18:30 light; 18:30-06:30 dark), with constant room temperature (21 ± 1 °C) and relative humidity (40-60 %). 2 guinea pigs are placed in a cage. The cages contained 1-1.5 cm layer of animal bedding. Animals had access to food and water ad libitum.
Wild animals	The study did not involve wild animals studies.

Field-collected samples	The study did not involve filed-collection samples.
Ethics oversight	All animal studies were approved by the Institutional Animal Care and Use Committee (IACUC) of the Animal Experiment Center of Wuhan University (Wuhan, China). All mouse experimental procedures were carried out following the Regulations for the Administration of Affairs Concerning Experimental Animals approved by the State Council of People's Republic of China.

Note that full information on the approval of the study protocol must also be provided in the manuscript.

Flow Cytometry

Plots

Confirm that:

- [x] The axis labels state the marker and fluorochrome used (e.g. CD4-FITC).
- [x] The axis scales are clearly visible. Include numbers along axes only for bottom left plot of group (a 'group' is an analysis of identical markers).
- [x] All plots are contour plots with outliers or pseudocolor plots.
- [x] A numerical value for number of cells or percentage (with statistics) is provided.

Methodology

Sample preparation	For FACS analysis, DCs cells were blocked by anti-CD16/32 antibody and then stained with anti-mouse CD11c, anti-mouse CD80 and anti-mouse CD86 (mature DCs: CD11c+CD80+CD86+). Macrophages were blocked with anti-CD16/32 antibody and then stained with anti-mouse CD11b, anti-mouse CD80 and anti-mouse CD206 antibodies (M1 phenotype: CD11b+CD80+CD206-, M2 phenotype: CD11b+CD80+CD206+).
Instrument	Facscalibur (BD, Accuri C6)
Software	FlowJo_V10
Cell population abundance	No post-sort fractions were collected through the Flow cytometry.
Gating strategy	The gating strategy was detailly exhibited in Figure S11.

- [x] Tick this box to confirm that a figure exemplifying the gating strategy is provided in the Supplementary Information.
